# C−X (X = N, O) Cross-Coupling Reactions Catalyzed by Copper-Pincer Bis(N-Heterocyclic Carbene) Complexes

**DOI:** 10.3389/fchem.2019.00012

**Published:** 2019-01-31

**Authors:** Jennifer L. Minnick, Doaa Domyati, Rachel Ammons, Laleh Tahsini

**Affiliations:** Department of Chemistry, Oklahoma State University, Stillwater, OK, United States

**Keywords:** Cu-pincer NHC complexes, Ullmann-type C–X coupling, air-assisted cross-coupling, copper-oxygen catalysts, N-aryl imidazoles, biarylethers

## Abstract

Over the last two decades, N-heterocyclic carbene (NHC)–copper catalysts have received considerable attention in organic synthesis. Despite the popularity of copper complexes containing monodentate NHC ligands and recent development of poly(NHC) platforms, their application in C–C and C–heteroatom cross-coupling reactions has been limited. Recently, we reported an air-assisted Sonogashira-type cross-coupling catalyzed by well-defined cationic copper-pincer bis(NHC) complexes. Herein, we report the application of these complexes in Ullmann-type C–X (X = N, O) coupling of azoles and phenols with aryl halides in a relatively short reaction time. In contrast to other well-defined copper(I) catalysts that require an inert atmosphere for an efficient C–X coupling, the employed Cu(I)-pincer bis(NHC) complexes provide good to excellent yields in air. The air-assisted reactivity, unlike that in the Sonogashira reaction, is also affected by the base employed and the reaction time. With Cs_2_CO_3_ and K_2_CO_3_, the oxygen-generated catalyst is more reactive than the catalyst formed under argon in a short reaction time (12 h). However, the difference in reactivity is compromised after a 24 h reaction with K_2_CO_3_. The efficient pincer Cu-NHC/O_2_/Cs_2_CO_3_ system provides great to excellent cross-coupling yields for electronically diverse aryl iodides and imidazole derivatives. The catalyst scope is controlled by a balance between nucleophilicity, coordinating ability, and the steric hindrance of aryl halides and N-/O-nucleophiles.

## Introduction

The N-arylazoles and biarylethers are among the most commonly found motifs in pharmaceuticals, biologically active molecules, crop-protection chemicals, and material science. A huge number of drugs contain N-arylimidazole moieties including cyclic AMP phosphodiesterase inhibitors (Sircar et al., 1987; Venuti et al., 1988; Güngör et al., 1992; Martinez et al., 1992; Sawanishi et al., 1997), thromboxane synthase inhibitors (Iizuka et al., 1981; Martinez et al., 1992; Cozzi et al., 1993; Nicolaï et al., 1993), topical antiglaucoma agents (Lo et al., 1992), and cardiotonic agents (Hagedorn et al., 1987; Erhardt et al., 1989; Shaw et al., 1992). In this regard, transition-metal catalyzed C-heteroatom coupling reactions have become one of the most important areas in modern chemical synthesis. Several methods have been developed for the direct coupling of azole with functionalized arenes. The most common route is nucleophilic aromatic substitution of azoles with aryl halides; however, this approach is limited to aryl halides with strongly electron-withdrawing groups (Venuti et al., 1988; Güngör et al., 1992; Cozzi et al., 1993; Ohmori et al., 1996). A second approach to N-arylazoles is traditional Ullmann-type coupling that has a broader aryl halide scope, but requires high temperatures (>150°C), polar solvents, and often stoichiometric amounts of copper or copper salts (Ullmann and Bielecki, 1901; Ullmann, 1903; Ullmann and Sponagel, 1905). To achieve milder C–N/C–O coupling conditions, efforts have focused on the use of more activated arenes such as arylbismuth (Barton et al., 1988), -tin, (Davydov et al., 2002) -lead (Lopez-Alvarado et al., 1995), -silanes (Lam et al., 2000), and -boronic acids (Lam et al., 1998; Mederski et al., 1999; Collot et al., 2000). Some of these methods are limited in scope to certain azole and arenes or produce toxic side-products, e.g., copper-catalyzed N-phenylation of indoles by triphenylbismuth bis(trifluoroacetate) or synthesis of N-arylimidazoles using *p*-tolyllead triacetate and Cu(OAc)_2_ catalyst (Barton et al., 1988; Lopez-Alvarado et al., 1995). A significant change to the conditions and the scope of azole N-arylation was made by Chan and Lam that reported direct coupling of arylboronic acids and azoles using stoichiometric amounts of Cu(OAc)_2_ and pyridine (or trimethylamine) at room temperature (Lam et al., 1998). A similar approach was alternatively used to synthesize diaryl ethers from phenols and arylboronic acids (Evans et al., 1998). The shortcoming of this method, use of stoichiometric amounts of copper salts and base, was later improved in the report of a diamine-copper-catalyzed N-arylation of imidazoles (Collman and Zhong, 2000). The scope has been recently extended to heterocycles and diarylethers (Lam et al., 2001; Guillou et al., 2009; Wentzel et al., 2009; Liu et al., 2010; Wang et al., 2010).

Another approach toward milder C(aryl)–heteroatom bond formation is via a ligand-accelerated Ullmann-type coupling of aryl halides and nucleophiles at relatively lower temperatures than those used in Ullmann condensation. Buchwald reported the synthesis of various N-arylimidazoles using catalytic amounts of Cu(OTf)_2_·C_6_H_6_ and stoichiometric amounts of 1,10-phenanthroline and *trans*,*trans*-dibenzylideneacetone in xylenes at different temperatures (110–125°C) (Kiyomori et al., 1999). A vital step to this method, facilitated by soluble cuprous ions, (Paine, 1987) is ligand screening to identify the most effective platform. In this regard, systematic studies were undertaken on N-, O-, and P-donor ligands as well as copper sources which has led to the development of efficient catalysts for cross-coupling of N-heterocycles and phenols with aryl halides (Arterburn et al., 2001; Klapars et al., 2001; Antilla et al., 2002, 2004; Kelkar et al., 2002; Evano et al., 2008). In addition to the catalysts formed *in-situ* from copper salts and ligands, some well-defined copper catalysts have been identified for the N-arylation of azoles and O-arylation of phenols (Gujadhur and Venkataraman, 2001; Gujadhur et al., 2001; Choudary et al., 2005; Biffis et al., 2009). Interestingly, the reports of phosphine- and NHC-supported copper catalysts in this chemistry has been limited, unlike N- and O-donor systems. To the best of our knowledge, there has been only two reports of NHC ligands in Ullmann-type coupling wherein the catalytic activity of cationic trinuclear Cu(I)-tris(NHC) and neutral mononuclear [Cu(IPr)Cl] has been assessed (Tubaro et al., 2008; Biffis et al., 2009). The trinuclear complexes presented high reactivities in C–N, C–O, and C–C coupling reactions with a low catalyst loading.

**Figure 1:**
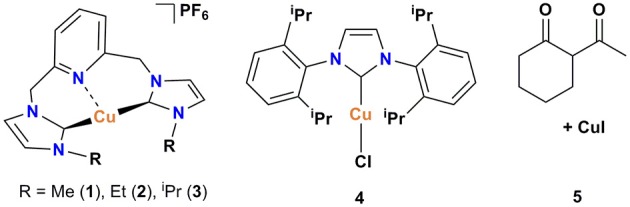
Copper catalysts used in this study.

Recently, we have developed mononuclear pyridylmethyl-linked Cu(I)-bis(NHC) complexes with alkyl wingtips, Cu-I(R)^C∧*N*∧*C*^, and examined their catalytic reactivity in an air-assisted cross-coupling of terminal alkynes and aryl iodides (Domyati et al., 2016, 2018). Our interest in Ullmann-type coupling was driven partly by a need to synthesize N-arylated imidazole derivatives for our pyridine-based bis(NHC) ligands with aryl wingtips. Herein, we report based-controlled, air-assisted Ullmann-type coupling of azoles and phenols with aryl halides facilitated by pincer Cu-NHC complexes (Figure 1).

## Results and Discussion

### Synthesis of Copper Catalysts

The pyridine-based pincer bis(NHC) complexes containing Me, Et, and ^i^Pr substituents were prepared via the reaction of tetrakis(acetonitrile)copper(I) hexafluorophosphate as copper source and an *in-situ* generated carbene following our previously described procedure (Domyati et al., 2016). To compare the catalytic activity of the cationic pincer Cu-NHC complexes to one of the most efficient copper-carbene catalysts known to date, [Cu(IPr)Cl] (**4**), it was prepared by modifying a published procedure (Santoro et al., 2013; Lazreg et al., 2015). In an attempt to conduct Ullmann-type coupling of azoles under ambient conditions similar to that reported previously for primary amines and N-heterocycles (Shafir and Buchwald, 2006), an *in-situ* formed copper catalyst from CuI and 2-acetylcyclohexanone (**5**) was also utilized.

### Cross-Coupling Reaction Optimization

We chose the coupling of imidazole and 4-iodoacetophenone as a model reaction to determine the optimal conditions for catalysis (Table 1). All reactions were carried out in a 20 mL screw-cap reaction vial that was charged with reagents and 5 mL reagent-grade solvent in air. The model reaction was initially performed using **3** as catalyst for 12 h in different solvents which provided the highest yield of N-arylimidazole in DMF at 120°C (Table 1, entry 5). The yield increased only to a slight extent (+4%) when the reaction time was doubled indicating an almost-completed cross-coupling in 12 h (Table 1, entry 6). This finding and the lack of any biaryl coupling, a potential side reaction in air, makes the pincer complexes one of the most efficient, well-defined catalysts for N-arylation of imidazole (Kiyomori et al., 1999; Biffis et al., 2009). The catalytic activity of the pincer Cu-NHC complexes is strictly controlled by temperature. Reducing the temperature to 60°C provided a much lower yield and no conversion occurred at room temperature (Table 1, entries 7-8). The model reaction was then tested using **1**, **2**, and **4** as other well-defined catalysts, however, the product was isolated at lower yields than that obtained with **3**. Interestingly, **4** provided the lowest yield, even lower than that of **3** at 5 mol% loading after 24 h (Table 1, entries 6, 11). The reduced catalytic activity of **4** in this reaction, compared to that reported previously for the same substrates in DMSO at 100°C, is most likely due to different solvent and/or atmosphere (Biffis et al., 2009). The cross-coupling reaction in the absence of a copper catalyst provided only 24% yield under the same reaction conditions (Table 1, entry 12). The optimal amount of the base was determined by the highest cross-coupling yield achieved for the model reaction using various amounts of Cs_2_CO_3_ (Supplementary Figure 1).

**Table 1:** Reaction of imidazole with 4-iodoacetophenone catalyzed by copper complexes^*a*^.

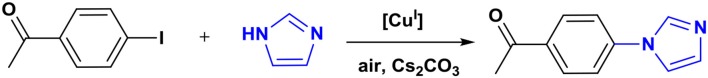					
**Entry**	**[Cu]**	**Solvent**	**Temp. (°C)**	**Time (h)**	**Yield (%)**^**b**^
1	3	DMSO	120	12	48
2	3	p-xylene	120	12	16
3	3	Dioxane	120	12	61
4	3	Toluene	120	12	<7
5	3	DMF	120	12	78
6	3	DMF	120	24	82 (62)*^c^*
7	3	DMF	60	24	33
8	3	DMF	r.t.	24	0
9	2	DMF	120	24	80
10	1	DMF	120	24	71
11	4	DMF	120	24	50
12	No copper	DMF	120	24	24
13	5	DMF	r.t.	24	63
14	5	DMF	120	24	77^d^ (78)^e^

a Reaction conditions were as follows: 4-iodoacetophenone (0.5 mmol), imidazole (0.75 mmol), Cs_2_CO_3_ (1.0 mmol), [Cu] (0.05 mmol), solvent (5 mL) in air.

b Isolated yield.

c **3** (0.025 mmol, 5 mol%) was used.

d Under Ar and using optimized amount of reagents in (Shafir and Buchwald, 2006).

e *Under air*.

Furthermore, we attempted to compare the pincer complexes activity to an *in-situ* formed catalyst, **5**, reported as an efficient catalyst for the Ullman-type coupling of primary amines and N-heterocycles (Shafir and Buchwald, 2006). First, the model N-arylation of imidazole was performed using **5** under the exact same conditions reported for the coupling of non-azole substrates. At room temperature, the **5**-catalyzed reaction of 4-iodoacetophenone and imidazole afforded 63% cross-coupling yield, whereas no product was formed using **3** (Table 1, entries 8, 13). Although this result makes **5** a more reactive catalyst than **3**, a further study revealed its limited scope due to a substrate-dependent reactivity with other aryl halides (Supplementary Figure 2). The coupling of imidazole with 4-iodonitrobenzene provided 67% yield at room temperature, comparable to that of the model reaction, but no cross-coupled product was isolated for 4-(trifluoromethyl)iodobenzene. Further attempts to improve the yields by conducting these reactions at a higher temperature (120°C) revealed a moderate increase for the model substrate (Table 1, entry 14). However, a reverse temperature effect was found with the 4-NO_2_ derivative and the conversion was reduced significantly. Additionally, temperature had no improving effect on the yield of 4-CF_3_ substrate and no conversion occurred even at 120°C. Unlike pincer Cu-NHC catalysts, air did not have a noticeable effect on the cross-coupling reactions catalyzed by **5** and no clear improvement of the yield was found (Table 1, entry 14).

### Atmosphere and Base Effect

**Figure 2:**
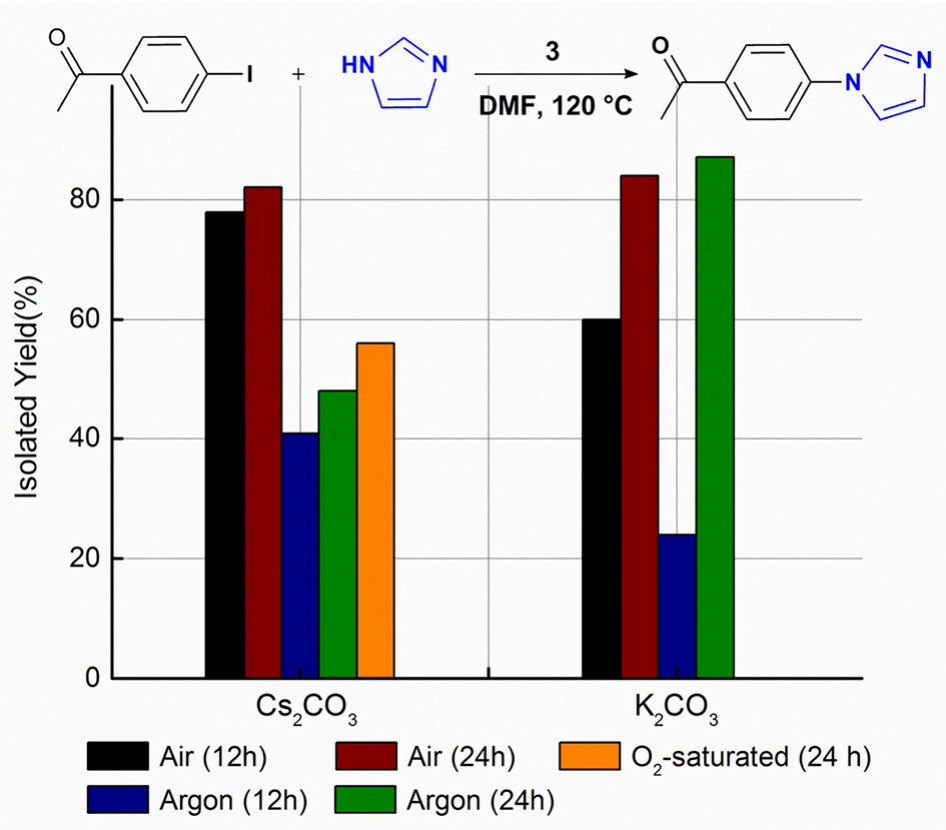
The isolated yield of the cross-coupled product from imidazole and 4-iodoacetophenone reaction catalyzed by **3** and Cs_2_CO_3_ or **3** and K_2_CO_3_ in DMF at 120°C under an air, O_2_-saturated, and argon atmosphere. Reaction conditions were as follows: aryl halide (0.5 mmol), imidazole (0.75 mmol), base (1.0 mmol), **3** (0.05 mmol), DMF (5 mL).

The assistant role of oxygen in Cu-NHC catalyzed Sonogashira reaction was previously shown by the greater yield of the product in air than that under argon (Domyati et al., 2018). This effect in Ullmann-type coupling was examined by conducting the model reaction under argon using the same optimized conditions found in air. After a 12 h anaerobic reaction, the N-arylimidazole was isolated at almost half of the yield obtained under air indicating the facilitating effect of oxygen in the C–N coupling reactions catalyzed by **3** as well (Figure 2). Interestingly, extending the reaction time to 24 h improved the yield only to a slight extent, similar to the trend observed for the aerobic reaction. To examine the effect of oxygen concentration, the model reaction was performed in O_2_-saturated DMF which provided a lower yield than in air but slightly more than that under argon. This indicates the significance of a certain stoichiometry of oxygen and copper to the catalytic activity, similar to that in **3**-catalyzed Sonogashira-type coupling (Domyati et al., 2018).

The oxygen-assisted cross-coupling mechanism is also controlled by the base used in the reaction. While Cs_2_CO_3_ provided more cross-coupled product in air than under argon in both 12 h and 24 h reaction times, the yields obtained with K_2_CO_3_ after 24 h were almost identical under both atmospheres. In contrast, K_2_CO_3_ provided a much smaller cross-coupling yield under argon in 12 h than in air supporting a slow formation of the active catalyst under anaerobic conditions. The distinct reactivity of Cu-I(R)^C∧*N*∧*C*^ complexes in air from that under argon could be due to different active catalysts and potentially a different mechanism that needs to be clarified. However, the presented data support a higher cross-coupling reactivity of the air-generated catalyst in the presence of Cs_2_CO_3_ than that in the presence of K_2_CO_3_. On the other hand, the slowly-formed active catalyst under argon is more reactive in the presence of K_2_CO_3_ than with Cs_2_CO_3_. Such versatility of active catalysts controlled by simple change of the base or atmosphere is significant to the application scope of the system.

### Reaction Scope

**Scheme 1:**
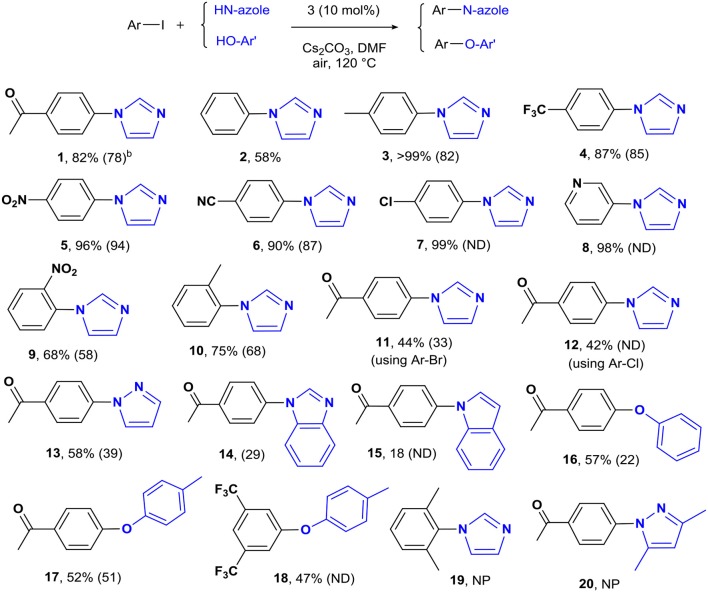
The coupling of aryl halides and N- and O-nucleophiles catalyzed by **3**^*a*^. ^a^Reaction conditions: aryl halide (0.5 mmol), imidazole (0.75 mmol), Cs_2_CO_3_ (1.0 mmol), [Cu] (0.05 mmol), solvent (5 mL), in air, 24 h. ^b^Isolated yields after 24 h reaction time. The yields in parentheses are after 12 h. ND, not determined; NP, no product.

The optimized conditions were applied to a range of electronically diverse aryl halides and different N- and O-nucleophiles to explore the application scope of the pincer Cu-NHC catalysts in Ullmann-type coupling. Considering the close yields obtained for the model reaction after 12 and 24 h, most reactions were performed at both times to examine any substrate-dependent behavior in this matter. The cross-coupled products were isolated with <10% difference in yield between the two reaction times, except for the reaction of 4-iodotoluene with imidazole and 4-iodoacetophenone with pyrazole and phenol (Scheme 1, **3**, **13**, **16**). The coupling of *p*-substituted aryl iodides bearing electron-donating and electron-withdrawing groups with imidazole provided the corresponding N-arylimidazoles in great (82%) to excellent (>99%) yields (Scheme 1). An exception to this trend is the coupling of unactivated iodobenzene and imidazole that provided a moderate yield (58%) of the product in 24 h (Scheme 1, **2**). The less pronounced effect of the directing groups of aryl halides on the reactivity of **3** is also shown by the comparable yields of *o*-substituted substrates. While the steric hindrance of 2-iodotoluene and 2-iodonitrobenzene led to a lower cross-coupled product than their *p*-substituted analogs, their isolated yields are close with slightly higher activity of 2-iodotoluene (Scheme 1, **9**, **10**). The steric effects appear to play a major role in the reactivity of aryl iodides in the present system given that no product was isolated from the reaction of 2,6-dimethyliodobenzene and imidazole (Scheme 1, **19**). The other important parameter affecting the catalyst reactivity is the type of halide. While aryl iodides are highly reactive using **3**, the coupling of activated aryl bromides and chlorides with imidazole was achieved at moderate yields (Scheme 1, **11, 12**). Additionally, the coupling of imidazole with heteroaryl iodides is facilitated given the excellent yield obtained for 3-iodopyridine (Scheme 1, **8**). The application scope of pincer Cu-NHC complexes in Ullmann-type coupling is also controlled by the type of nucleophile used. The cross-coupling of 4-iodoacetophenone with exemplary heterocycles such as indole, pyrazole, and benzimidazole, as well as phenols provided low (18%) to moderate yields (58%) of corresponding products (Scheme 1, **13**–**18**). The reactivity pattern of nucleophiles appears to be the result of a balance between nucleophilicity and coordinating ability to the copper center controlled by steric effects in the order: imidazole > pyrazole > phenol > benzimidazole > indole. This is further confirmed by no cross-coupled product isolated from the reaction of 3,5-dimethylpyrazole and aryl iodide due to the greater steric hindrance of substituted pyrazole compared to pyrazole, despite its higher nucleophilicity (Scheme 1, **20**).

To clarify the scope of reactivity in O-arylation, we examined the coupling of exemplary phenol and aryl iodide derivatives bearing electron-donating and electron-withdrawing groups. Complex **3** showed good reactivity toward activated aryl iodides and phenols with moderate yields (47–57%) isolated from the phenol and p-cresol reaction with 4-iodoacetophenone and 3,5-bis(trifluoromethyl)-iodobenzene (Scheme 1, **16**–**18**). Interestingly, electron-rich aryl halide and/or electron-poor phenols shut down the reaction completely such that no cross-coupled product was isolated.

### Comparison to Other Cu-NHC Catalysts

The pincer Cu-NHC complexes (**1**–**3**) present a new class of Ullmann-type C–heteroatom coupling catalysts with distinct structure and properties from those reported previously (Table 2). While complex **4** is air-stable and benefits from good solubility in less polar solvents due to charge neutrality, the Cu-triscarbene complexes are applicable at a low catalyst loading owing to three Cu(I) ions in a molecular unit. On the other hand, pincer Cu-NHC complexes bearing small alkyl wingtips and open coordination sites can react with oxygen leading to more efficient C–X coupling systems in air than under argon. We have conducted a comparative study between pincer complex **3** and other Cu-NHC systems by taking into account the N-nucleophile substrates used in common (Table 2).

**Table 2:** Catalytic activity of different Cu-NHC complexes in selected C–N coupling reactions^*a*^.

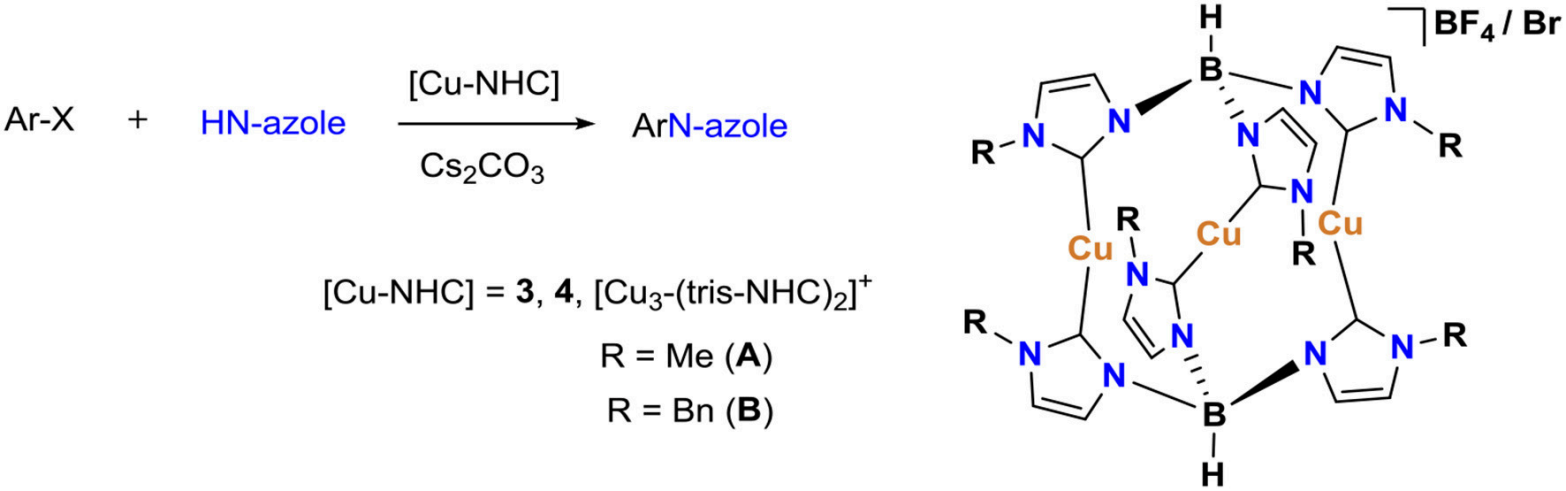						
**Entry**	**Aryl halide**	**Azole**	**Yield (%)**			
			**3**^**a**^	**A**^**b**^	**B**^**c**^	**4**^**c**^
1	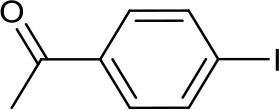	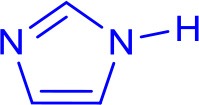	82	>99	85	86
2	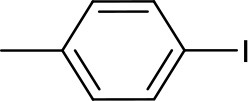	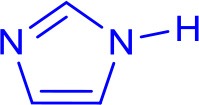	>99	20	93	ND
3	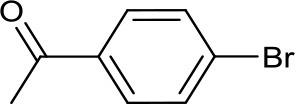	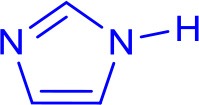	44	70	54	86
4	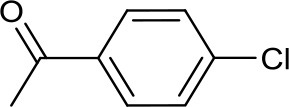	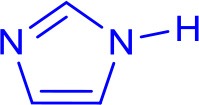	42	50	24	57
5	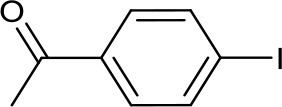	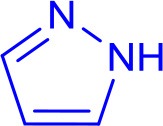	58 (62)^*d*^	93	ND	90

a Reaction conditions were as follows: aryl halide (0.5 mmol), imidazole (0.75 mmol), Cs_2_CO_3_ (1.0 mmol), [Cu] (0.05 mmol), solvent (5 mL) in air, 24 h.

b Literature data (see Tubaro et al., 2008).

c Literature data (see Biffis et al., 2009).

d *K_2_CO_3_ was used as a base*.

In contrast to **4** and the Cu-triscarbene with methyl wingtips (Table 2, **A**) that present a low reactivity toward aryl halides bearing weakly electron-donating groups, complex **3** is able to efficiently convert both activated, electron-poor aryl iodides and electron-rich ones (Table 2, entries 1, 2). In addition, **3** is able to facilitate the coupling of activated aryl bromides and chlorides with a comparable, moderate efficiency to the Cu-triscarbene complex **B**, but with a lower efficiency than that of complex **4** (Table 2, entries 3, 4). Regarding other azoles, **3** performs with a lower efficiency toward N-arylation of pyrazoles than other Cu-NHC complexes (Table 2, entry 5). To the best of our knowledge, the reactivity of other Cu-NHC complexes in cross-coupling of aryl halides with benzimidazole and indole substrates has not been reported.

## Materials and Methods

### General Procedure

Imidazolium salts and Cu(I) complexes (Figure 1, **1–3**) were synthesized according to published procedure using standard Schlenk techniques or in an MBraun glovebox under Ar atmosphere. 1,3-Bis(diisopropylphenyl)imidazolium chloride, IPr.HCl, was obtained from TCI Chemicals. The solvents used for the syntheses were purified and distilled under N_2_ atmosphere before being stored over activated molecular sieves (4 Å) in the glovebox. Tetrahydrofuran was purified by refluxing over Na/benzophenone under a N_2_ atmosphere and distilled. Hexane was distilled from CaH_2_ under N_2_. Reagent-grade N,N-dimethylformamide (DMF), 2-acetylcyclohexanone, cesium- and potassium carbonate, and copper iodide were purchased from Sigma-Aldrich and used as received. Aryl halides were obtained from TCI Chemicals and Sigma. Flash chromatography was performed on Merck silica gel 60 (230–400 mesh) obtained from Sigma-Aldrich. NMR spectra were measured using a 400 MHz Bruker Avance III spectrometer. NMR samples were prepared in deuterated solvents, CD_3_CN, CDCl_3_, or DMSO-d_6_ containing 0.05% TMS as internal standard. Chemical shifts (δ) for ^1^H and ^13^C NMR spectra were referenced to the resonance of TMS as an internal reference or the residual protio solvent. GC/MS data were collected using Shimadzu GC-MS-QP-2010S.

### Synthesis of [Cu(IPr)Cl], 4

The compound was synthesized by modifying a published procedure (Santoro et al., 2013). Under an argon atmosphere, CuCl (0.105 g, 1.06 mmol), IPr.HCl (0.300 g, 0.706 mmol), and K_2_CO_3_ (0.293 g, 2.12 mmol) were mixed in a reaction vial, and transferred out of the glovebox. The vial was quickly charged with 4–5 mL acetone and kept under refluxing conditions for 48 h. The reaction mixture was filtered through Celite and rinsed with 10–15 mL acetone and 6–7 mL dichloromethane. The filtrate was then concentrated under vacuum and layered with hexane. The resultant white solid was then filtered and kept under vacuum to dry completely. The solid was transferred into a glovebox and recrystallized from THF and hexane affording white crystals in 78% yield (0.270 g). ^1^H NMR (CD_3_CN with 0.05% v/v TMS, 400 MHz): δ = 7.56 (t, 1H; J = 7.8 Hz), 7.46–7.36 (m, 3H), 2.56 (septet, 2H; J = 6.9 Hz), 1.24 (dd, 12H; J = 6.9 Hz). ^13^C NMR (CD_3_CN with 0.05% v/v TMS, 101 MHz): δ = 146.94, 135.59, 131.47, 125.12, 124.84, 29.52, 29.33, 24.98, 23.86.

### Cross-Coupling Reactions Catalyzed by 3 and 4 in Air

A mixture of copper complex (0.05 mmol) and cesium carbonate (1 mmol) was added to a 20 mL vial charged with a Teflon stir bar in the glovebox. The mixture was transferred out of the glovebox and suspended in 2 mL non-anhydrous DMF. To the resulting yellow suspension, a solution of aryl halide (0.5 mmol) in 2 mL DMF followed by a solution of azole or phenol (0.75 mmol) in 1 mL DMF was added. The vial was then sealed and placed in an oil bath with pre-adjusted temperature at 120°C for 12 or 24 h. The cooled mixture was diluted with 20–30 mL ethyl acetate and filtered over a pad of silica. The silica was washed with 30–40 mL ethyl acetate and the filtrate was placed under vacuum until dry. The residual material was loaded on a silica gel column and eluted with mixtures of hexane and ethyl acetate to afford the corresponding product. The cross-coupling reactions were repeated twice to ensure reproducibility.

### Cu-Catalyzed Coupling of 4-Iodoacetophenone and Imidazole in O_2_-Saturated DMF

In an argon-filled glovebox, a 20 mL scintillation vial was charged with a Teflon stir bar, copper complex (0.05 mmol), cesium carbonate (1.0 mmol), 4-Iodoacetophenone (0.5 mmol), and imidazole (0.75 mmol). The vial was sealed with a teflon cap bearing a silicone septum and transferred out of the glovebox. To this mixture, 5 mL O_2_-saturated DMF prepared by bubbling dry O_2_ gas through the solvent for 20 min was added. The reaction vial was then placed in an oil bath with pre-adjusted temperature at 120°C. After reaction completion, the mixture was cooled down, diluted with 20 mL ethyl acetate, and filtered through a pad of silica. The silica was washed with 30–40 mL ethyl acetate and the solvent was then removed under vacuum. The residue was purified by column chromatography to obtain analytically pure product.

### Cross-Coupling Reactions Catalyzed by CuI and 2-Acetylcyclohexanone, 5

Under an argon atmosphere, a 20 mL scintillation vial was charged with a Teflon stir bar, CuI (0.05 mmol), cesium carbonate (2.0 mmol), aryl halide (1.0 mmol), imidazole (1.5 mmol), and 3.5 mL anhydrous DMF. To the mixture, 2-acetylcyclohexanone (0.2 mmol) and 0.5 mL DMF were then added. The vial was sealed, brought out of the glovebox and kept stirring at room temperature or was placed in an oil bath with pre-adjusted temperature at 110°C. The progress of the reaction was monitored by TLC at different time intervals. Upon completion of the reaction, the reaction mixture was cooled down, diluted with 20 mL ethyl acetate, and filtered through a pad of silica gel. The silica was washed with 30 mL ethyl acetate and the solvent was then removed under vacuum. The residue was diluted with 2–3 mL ethyl acetate and purified by column chromatography using varying gradients of hexane and ethyl acetate to obtain analytically pure product.

## Conclusion

We have developed a new protocol for the Ullmann-type coupling of aryl halides with N- and O-nucleophiles catalyzed by pincer Cu-NHC complexes bearing alkyl wingtips. Unlike other well-defined Cu catalysts that are more efficient under inert atmosphere, the pincer complexes present generally a higher reactivity in air. This could be due to different active catalysts involved in the rate-determining step of the aerobic and anaerobic reactions. The oxygen-generated active catalyst was found more reactive than the catalyst formed under argon as indicated by the higher cross-coupled product obtained in air than that under argon. The air-assisted reactivity is also affected by the type of the base in the Ullman-type coupling. The catalytic reaction in air was accelerated to a greater extent using Cs_2_CO_3_ than K_2_CO_3_ as indicated by a nearly-completed reaction and a higher cross-coupling yield achieved in 12 h. Building on these findings and by taking advantage of the air-assisted reactivity of pincer complexes, an efficient cross-coupling of aryl iodides and imidazole derivatives has been developed. The catalytic system performed with a great to excellent efficiency toward the coupling of electron-poor and electron-rich aryl iodides with imidazole, but had moderate reactivity with pyrazole and aryl bromides or aryl chlorides. Further studies to clarify the reactivity of the Cu-pincer NHC complexes with nucleophiles and to improve their efficiency toward C–O coupling reactions are currently ongoing in our group.

## Author Contributions

JM and DD designed and completed most of the experiments and helped with data analysis. RA helped with the synthesis of copper-NHC catalysts and some of the catalytic reactions. LT and JM co-wrote the manuscript and were responsible for discussing and revising the paper.

### Conflict of Interest Statement

The authors declare that the research was conducted in the absence of any commercial or financial relationships that could be construed as a potential conflict of interest.

## Supplementary Material

^1^H and ^13^C{^1^H} NMR spectra for **4** and cross-coupled products (Supplementary Figures 3–19), catalytic performance graph of CuI/α,β-diketone catalyst (**5**), optimization graph of the base amount, and GC/MS data of cross-coupled products.
