# Supplementary material for: C−X (X = N, O) Cross-Coupling Reactions Catalyzed by Copper-Pincer Bis(N-Heterocyclic Carbene) Complexes
Source: Front Chem. 2019 Jan 31;7:12. doi: 10.3389/fchem.2019.00012 (PMC6365418; doi:10.3389/fchem.2019.00012)
Supplement: Supplementary file 1 [file Data_Sheet_1.PDF]

Supplementary Material

C–X (X = N, O) Cross-Coupling Reactions Catalyzed by Copper-Pincer Bis(N-Heterocyclic Carbene) Complexes

Jennifer L. Minnick[1], Doaa Domyati[1], Rachel Ammons[1], Laleh Tahsini[1*]

*** Correspondence:** Dr. Laleh Tahsini: tahsini@okstate.edu

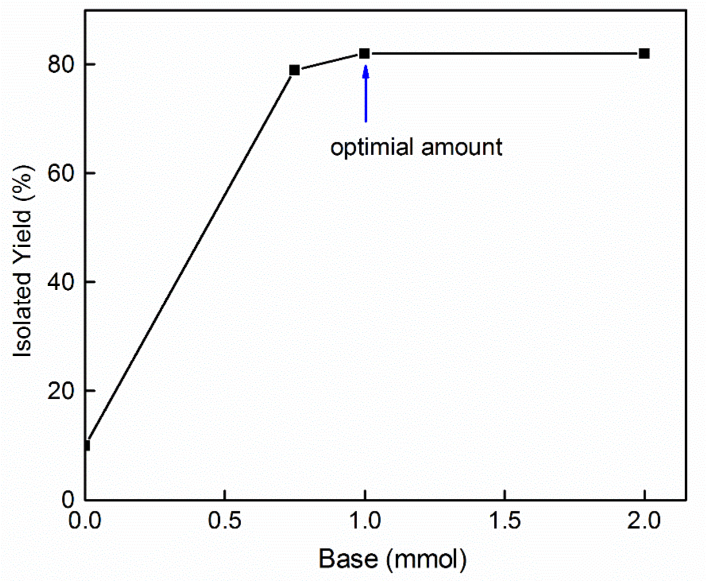

Supplementary Figure 1. The isolated yields of C–N cross-coupled products from the reaction of imidazole with 4-iodoacetophenone catalyzed by **3** in DMF at 120 °C under air. Reaction conditions: aryl halide (0.5 mmol), imidazole (0.75 mmol), Cs_2CO_3 (0.0-2.0 mmol), **3** (0.05 mmol), DMF (5 mL).

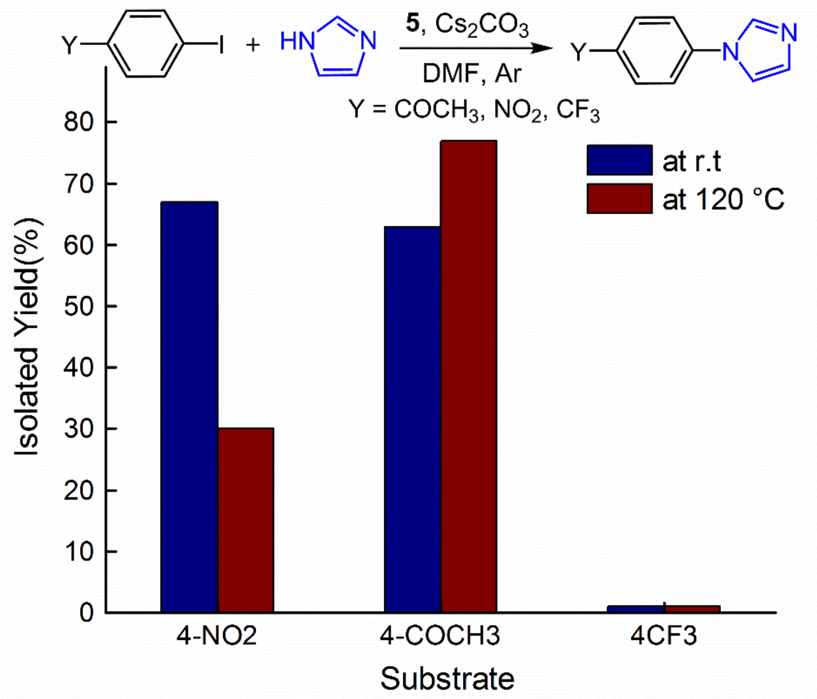

Supplementary Figure 2. The isolated yields of C–N cross-coupled products from the reaction of imidazole with 4-iodoacetophenone, 4-iodonitrobenzene, and 4-(trifluoromethyl)iodobenzene catalyzed by CuI and 2-acetylcyclohexanone (**5**) in DMF at room temperature and 120 °C under argon in 24 h. Reaction conditions: aryl halide (1.0 mmol), imidazole (1.5 mmol), Cs_2CO_3 (2.0 mmol), CuI (0.05 mmol), diketone (0.2 mmol), solvent (4 mL).

Ullmann-Type Cross-Coupling Products. The following cross-coupled products were prepared according to the general procedure described previously.

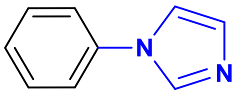

1-phenyl-1H-imidazole

Following the general procedure, the reaction of 4-iodobenzene and 1*H*-imidazole provided the isolated product as an off-white solid (58%) after purification on a pad of silica gel column with a gradient of hexane/ethyl acetate. ^{1}HNMR ($CDCl_3$, 400MHz) δ 7.78 (d, 2H), 7.48 (t, 2H), 7.44-7.32 (m, 3H), 7.32-6.91 (m, 1H); GC/MS *m/z* 144 (19.5 min).

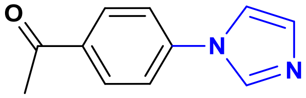

1-(4-Imidazol-1-yl-phenyl)-ethanone

Following the general procedure, the reaction of 4-iodoacetophenone and 1*H*-imidazole provided the isolated product as an off-white solid (78%) after purification on a pad of silica gel column with a gradient of hexane/ethyl acetate. ^{1}HNMR ($CDCl_3$, 400MHz) δ 8.10-8.07 (d, 2H), 7.95 (s, 1H), 7.51-7.48 (d, 2H), 7.35 (s, 1H), 7.24 (s, 1H), 2.64 (s, 1H); ^{13}CNMR ($CDCl_3$, 101 MHz) δ 196.64, 140.87, 135.90, 135.50, 131.31, 130.46, 120.84, 117.83, 26.73; GC/MS *m/z* 186 (21.7 min).

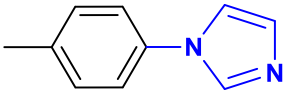

1-(4-Methylphenyl)-1*H*-imidazole

Following the general procedure, the reaction of 4-iodotoluene and 1*H*-imidazole provided the isolated product as a yellow oil (>99%) after purification on a silica gel column with a gradient of hexane/ethyl acetate. ^{1}HNMR (DMSO, 400MHz) δ 8.20 (s, 1H), 7.69 (s, 1H), 7.53-7.50 (d, 2H), 7.32-7.30 (d, 2H), 7.08 (s, 1H), 2.34 (s, 3H); ^{13}CNMR (DMSO, 101 MHz) δ 136.24, 135.40, 134.60, 130.20, 129.71, 120.21, 117.98, 20.42; GC/MS *m/z* 158 (17.6 min).

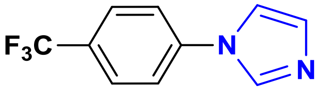

1-[4-(Trifluoromethyl)phenyl]-1*H*- imidazole

Following the general procedure, the reaction of 4-iodobenzotrifluoride and 1*H*-imidazole provided the isolated product as an off-white solid (87%) after purification on a silica gel column with a gradient of hexane/ethyl acetate. ^{1}HNMR ($CDCl_3$, 400MHz) δ 7.92 (s, 1H), 7.77-7.75 (d, 2H), 7.54-7.52 (d, 2H), 7.33 (s, 1H), 7.25 (s, 1H); ^{13}CNMR ($CDCl_3$, 101 MHz) δ 140.14, 135.57, 131.35, 129.85, 129.52, 127.40, 125.12, 122.42, 121.43, 117.99; GC/MS *m/z* 212 (15.9 min).

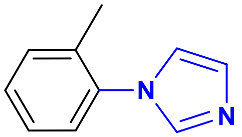

1-(2-Methylphenyl)-1*H*-imidazole

Following the general procedure, the reaction of 2-iodotoluene and 1*H*-imidazole provided the isolated product as a yellow oil (75%) after purification on a silica gel column with a gradient of hexane/ethyl acetate. ^{1}HNMR (DMSO, 400MHz) δ 7.82 (s, 1H), 7.43-7.28 (m, 5H), 7.09 (s, 1H), 2.15 (s, 1H); ^{13}CNMR (DMSO, 101 MHz) δ 137.65, 136.56, 133.13, 131.19, 128.71, 128.46, 126.94, 126.29, 120.88, 17.41; GC/MS *m/z* 158 (16.2 min).

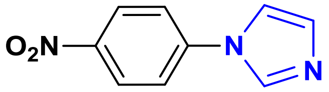

1-(4-Nitrophenyl)-1*H*-imidazole

Following the general procedure, the reaction of 1-iodo-4-nitrobenzene and 1*H*-imidazole provided the isolated product as a yellow solid (94%) after purification on a silica gel column with a gradient of hexane/ethyl acetate. ^{1}HNMR ($CDCl_3$, 400MHz) δ 8.43-8.35 (m, 2H), 7.99 (s, 1H), 7.63-7.54 (m, 2H), 7.38 (s, 1H), 7.29 (s, 1H); ^{13}CNMR ($CDCl_3$, 101 MHz) δ 146.45, 142.12, 135.50, 131.86, 125.93, 121.23, 117.93; GC/MS *m/z* 189 (22.3 min).

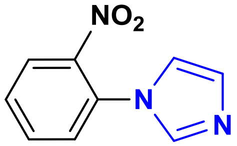

1-(2-Nitrophenyl)-1*H*-imidazole

Following the general procedure, the reaction of 1-iodo-2-nitrobenzene and 1*H*-imidazole provided the isolated product as a yellow solid (68%) after purification on a silica gel column with a gradient of hexane/ethyl acetate. ^{1}HNMR ($CDCl_3$, 400MHz) δ 8.01-7.99 (d, 1H), 7.76-7.71 (t, 1H), 7.65-7.60 (t, 2H), 7.48-7.46 (d, 1H), 7.22 (s, 1H), 7.07 (s, 1H); ^{13}CNMR ($CDCl_3$, 101 MHz) δ 145.44, 134.70, 133.89, 130.75, 130.49, 129.81, 128.80, 125.49, 123.58; GC/MS *m/z* 189 (20.2 min).

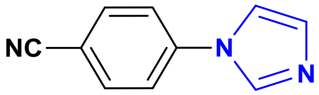

4-Imidazole-1-yl-benzonitrile

Following the general procedure, the reaction of 4-iodobenzonitrile and 1*H*-imidazole provided the isolated product as a yellow solid (90%) after purification on a silica gel column with a gradient of hexane/ethyl acetate. ^{1}HNMR ($CDCl_3$, 400MHz) δ 7.93 (s, 1H), 7.82-7.78 (d, 2H), 7.55-7.52 (d, 2H), 7.33 (s, 1H), 7.25 (s, 1H); ^{13}CNMR ($CDCl_3$, 101 MHz) δ 140.63, 134.26, 131.68, 121.49, 117.96, 111.23; GC/MS *m/z* 169 (20.7 min).

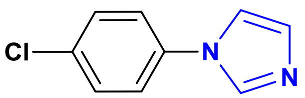

1-(4-Chloro-phenyl)-1*H*-imidazole

Following the general procedure, the reaction of 1-chloro-4-iodobenzene and 1*H*-imidazole provided the isolated product as a pale yellow solid (>99%) after purification on a silica gel column with a gradient of hexane/ethyl acetate. ^{1}HNMR (DMSO, 400MHz) δ 8.28 (s, 1H), 7.77 (s, 1H), 7.72-7.69 (d, 2H), 7.60-7.56 (d, 2H), 7.11 (s, 1H); ^{13}CNMR (DMSO, 101 MHz) δ 135.60, 135.43, 130.82, 129.89, 129.55, 121.78, 117.81; GC/MS *m/z* 178 (18.6 min).

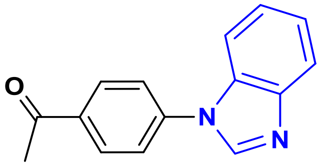

1-(4-(1*H*-benzo[*d*]imidazol-1-yl)phenyl)ethan-1-one

Following the general procedure, the reaction of 4-iodoacetophenone and benzimidazole provided the isolated product as an off-white solid (29%) after purification on a silica gel column with a gradient of hexane/ethyl acetate. ^{1}HNMR ($CDCl_3$, 400MHz) δ 8.20-8.18 (d, 3H), 7.92-7.89 (m, 1H), 7.67-7.64 (d, 2H), 7.63-7.58 (m, 1H), 7.40-7.37 (m, 2H), 2.69 (s, 3H); ^{13}CNMR ($CDCl_3$, 101 MHz) δ 196.73, 144.44, 141.94, 140.35, 136.27, 133.21, 130.50, 124.29, 123.48, 123.41, 121.05, 110.56, 26.81; GC/MS *m/z* 236 (27.2 min).

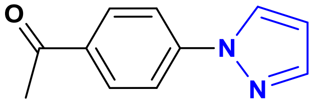

1-(4-(1*H*-pyrazol-1-yl)phenyl)ethan-1-one

Following the general procedure, the reaction of 4-iodoacetophenone and pyrazole provided the isolated product as a pale orange solid (58%) after purification on a silica gel column with a gradient of hexane/ethyl acetate. ^{1}HNMR ($CDCl_3$, 400MHz) δ 8.08-8.05 (d, 2H), 8.02-8.01 (d, 1H), 7.83-7.80 (d, 2H), 7.78-7.77 (d, 1H), 6.53-6.52 (t, 1H); ^{13}CNMR ($CDCl_3$, 101 MHz) δ 196.95, 143.43, 142.17, 134.90, 130.12, 126.99, 118.50, 108.69, 26.71; GC/MS *m/z* 186 (20.4 min).

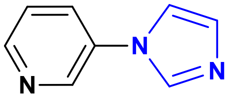

3-(1*H*-imidazol-1-yl)pyridine

Following the general procedure, the reaction of 3-iodopyridine and 1*H*-imidazole provided the isolated product as a yellow liquid (98%) after purification on a silica gel column with a gradient of hexane/ethyl acetate. ^{1}HNMR ($CDCl_3$, 400MHz) δ 8.74 (m, 1H), 8.63 (m, 1H), 7.88 (bs, 1H), 7.46-7.42 (m, 1H), 7.31 (bs, 2H), 7.27 (s, 1H); ^{13}CNMR ($CDCl_3$, 101 MHz) δ 148.94, 135.05, 143.05, 134.07, 131.33, 128.98, 124.42, 120.77.

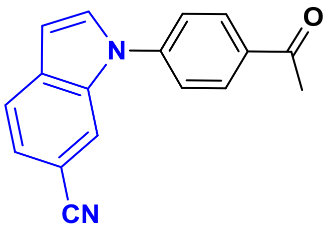

1-(4-acetylphenyl)-1H-indole-6-carbonitrile

Following the general procedure, the reaction of 4-iodoacetophenone and 6-cyanoindole provided the desired product as an orange solid (18%) after purification on a silica gel column with a gradient of hexane/ethyl acetate. ^{1}H NMR ($CDCl_3$, 400MHz) δ 8.18-8.16 (d, 2H), 7.91 (s, 1H), 7.70-7.65 (m, 3H), 7.60-7.58 (d, 2H), 6.81 (s, 1H), 2.69 (s, 3H).

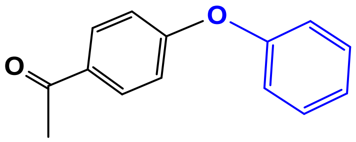

1-(4-Phenoxyphenyl)ethan-1-one

Following the general procedure, the reaction of 4-iodoacetophenone and phenol gave the desired product as an orange oil (57%) after purification on a silica gel column with a gradient of hexane/ethyl acetate. ^{1}HNMR ($CDCl_3$, 400MHz) δ 7.95-7.93 (d, 2H), 7.42-7.38 (t, 1H), 7.25-7.18 (t, 1H), 7.08-7.05 (d, 2H), 7.02-6.98 (d, 2H), 2.57 (s, 3H); ^{13}CNMR ($CDCl_3$, 101 MHz) δ 197.03, 162.15, 155.59, 131.98, 130.75, 130.19, 124.76, 120.31, 117.41, 26.59; GC/MS *m/z* 212 (21.3 min).

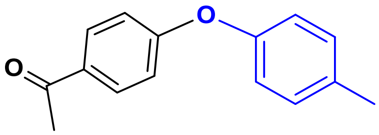

1-(4-(p-Tolyloxy)phenyl)ethan-1-one

Following the general procedure, the reaction of 4-iodoacetophenone and p-cresol gave the desired product as an orange oil (52%) after purification on a silica gel column with a gradient of hexane/ethyl acetate. ^{1}HNMR ($CDCl_3$, 400MHz) δ 7.94-7.91 (d, 2H), 7.22-7.18 (d, 2H), 6.98-6.95 (m, 4H), 2.57 (s, 3H), 2.37 (s, 3H); ^{13}CNMR ($CDCl_3$, 101 MHz) δ 197.39, 162.70, 153.58, 131.60, 130.77, 130.68, 129.86, 120.36, 116.98, 26.56, 20.94; GC/MS *m/z* 226 (22.6 min).

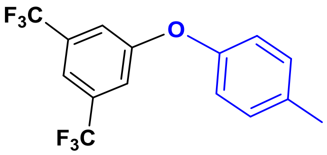

1-(p-Tolyloxy)-3,5-bis(trifluoromethyl)benzene

Following the general procedure, the reaction of 1-iodo-3,5-bis(trifluoromethyl)benzene and p-cresol gave the desired product as a yellow oil (47%) after purification on a silica gel column with a gradient of hexane/ethyl acetate. ^{1}HNMR ($CDCl_3$, 400MHz) δ 7.54 (s, 1H), 7.35 (s, 2H), 7.24-7.22 (d, 2H), 6.97-6.94 (d, 2H), 2.39 (s, 3H); ^{13}CNMR ($CDCl_3$, 101 MHz) δ 159.40, 152.66, 135.20, 133.38, 133.05, 131.04, 124.48, 121.77, 120.09, 20.97; GC/MS *m/z* 320 (15.1 min).

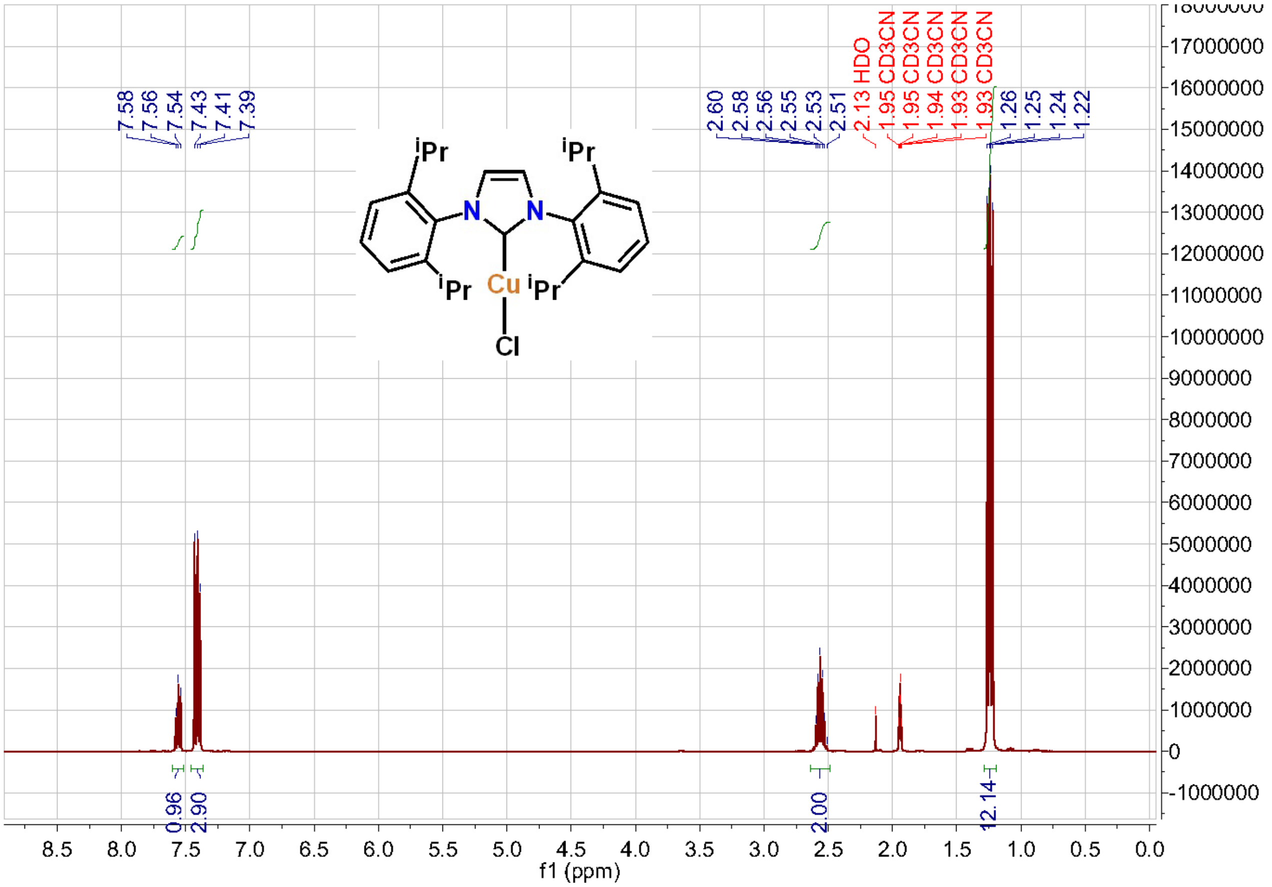

Supplementary Figure 3. ^{1}H NMR spectrum of [Cu(IPr)Cl] complex

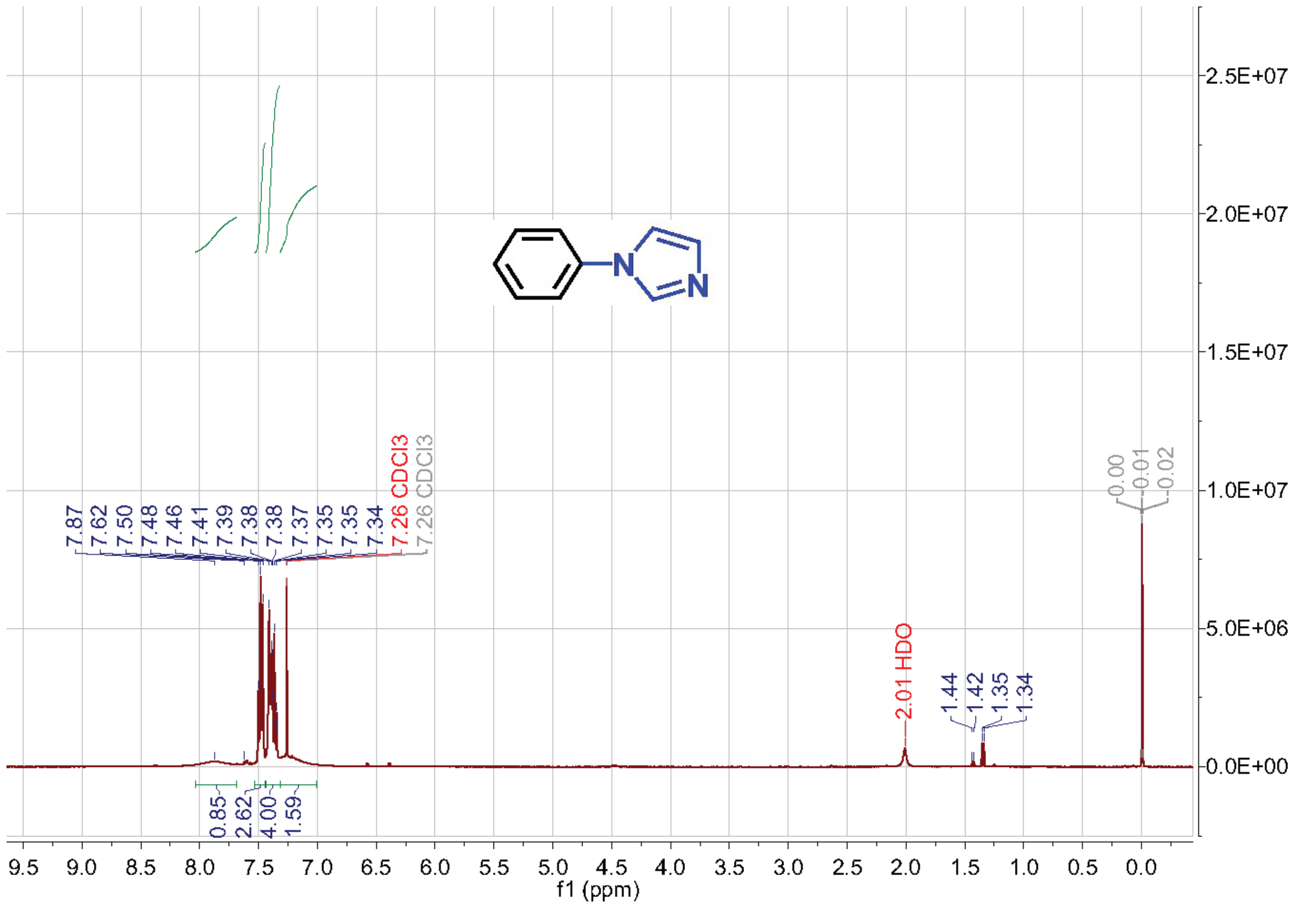

Supplementary Figure 4. ^{1}H MR spectrum of 1-phenyl-1H-imidazole, Scheme 1, **1**.

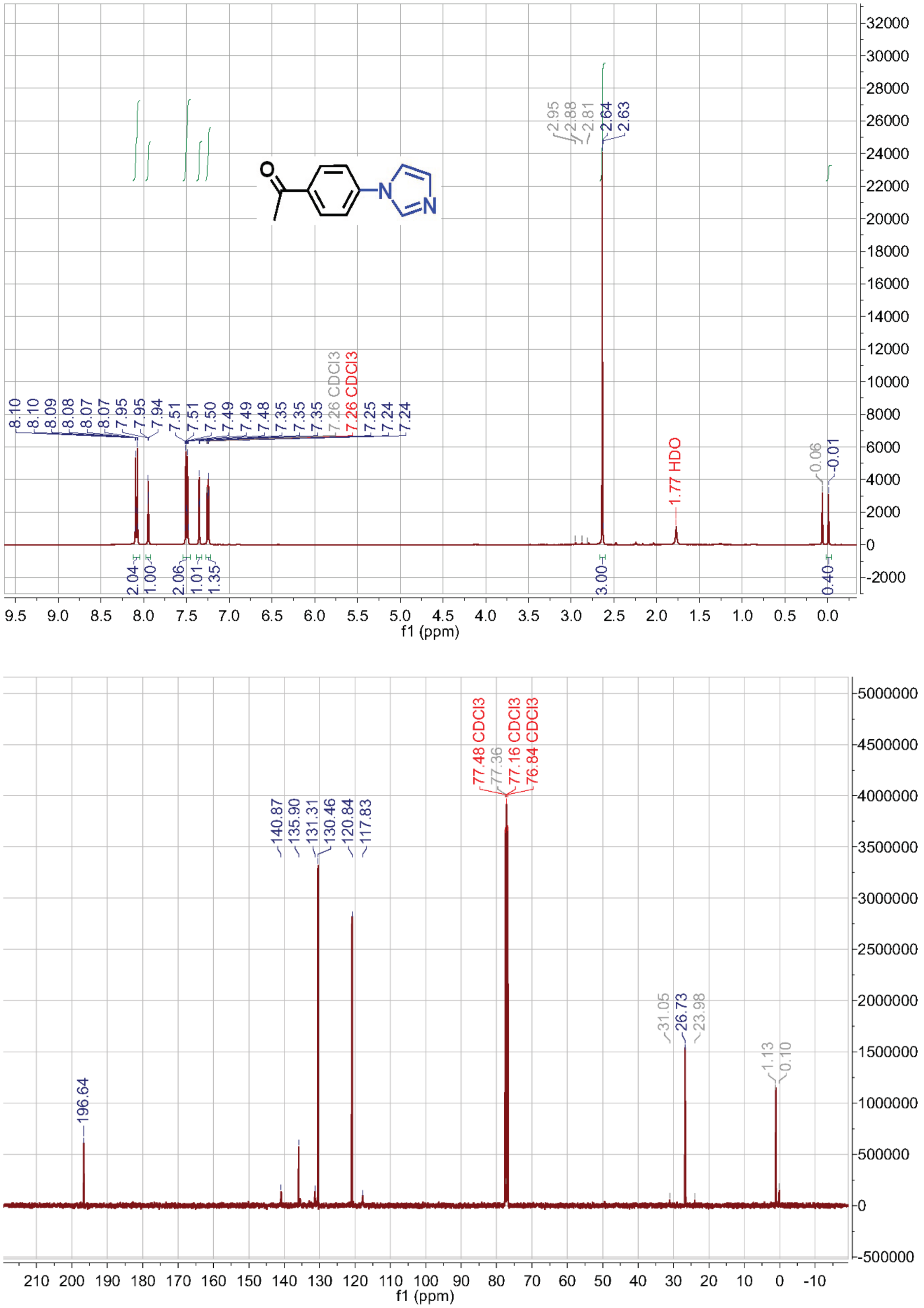

Supplementary Figure 5. ^{1}H MR and ^{13}C NMR spectra of 1-(4-Imidazol-1-yl-phenyl)-ethanone, Scheme 1, **2**.

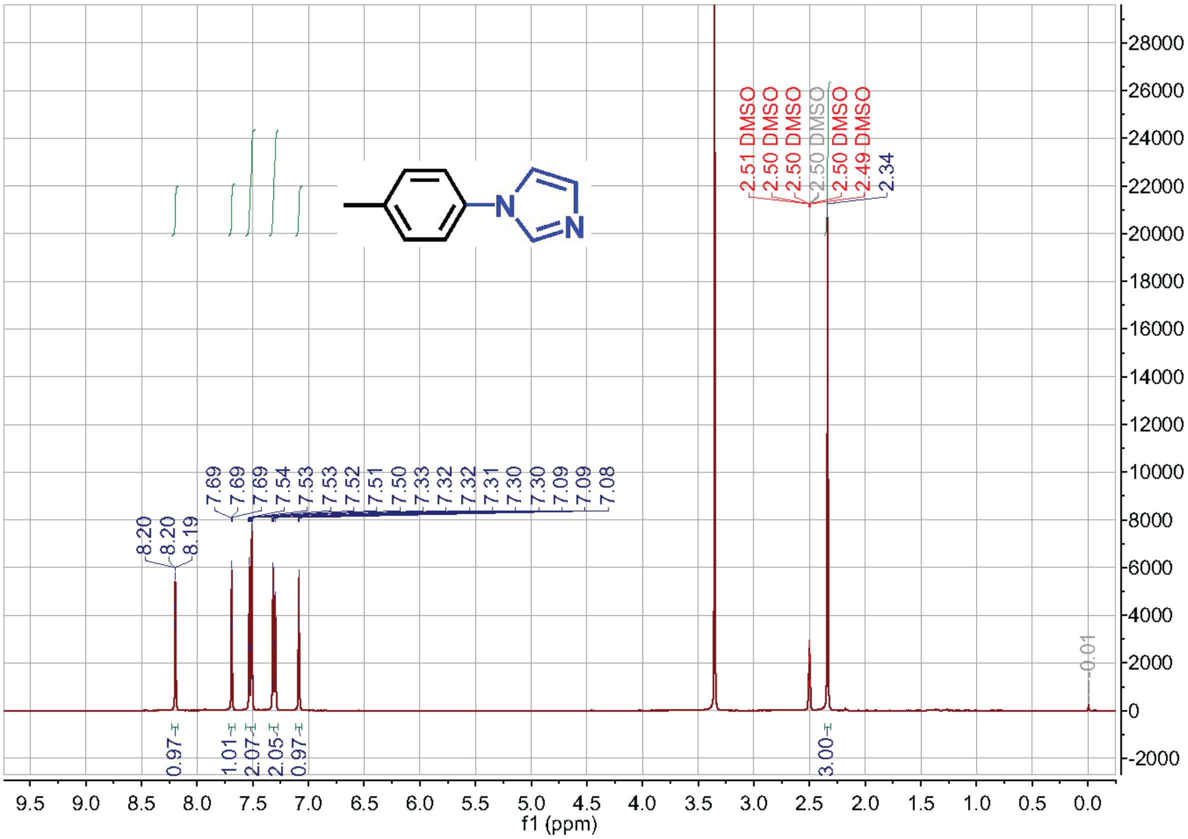

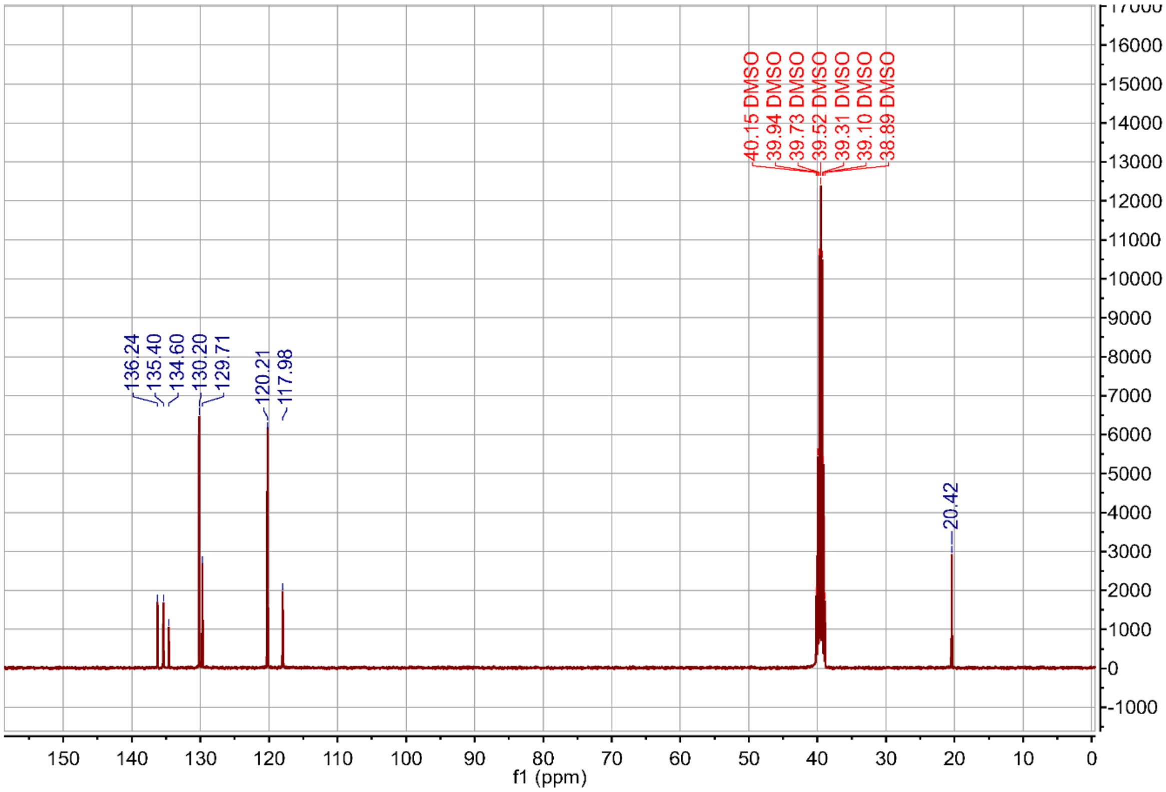

Supplementary Figure 6. ^{1}H MR and ^{13}C NMR spectra of 1-(4-Methylphenyl)-1H-imidazole, Scheme 1, **3**.

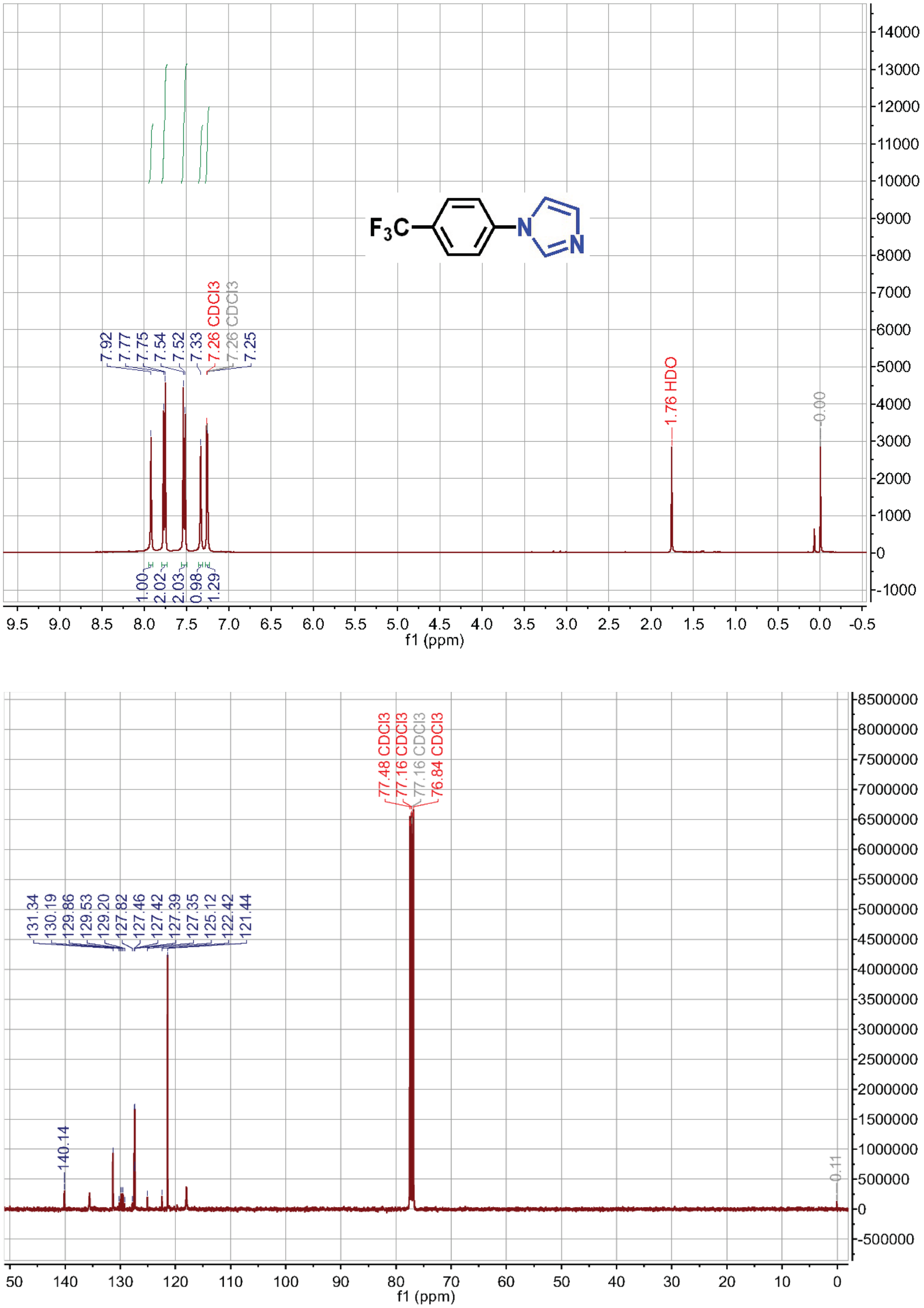

Supplementary Figure 7. ^{1}H MR and ^{13}C NMR spectra of 1-[4-(Trifluoromethyl)phenyl]-1H-imidazole, Scheme 1, **4**.

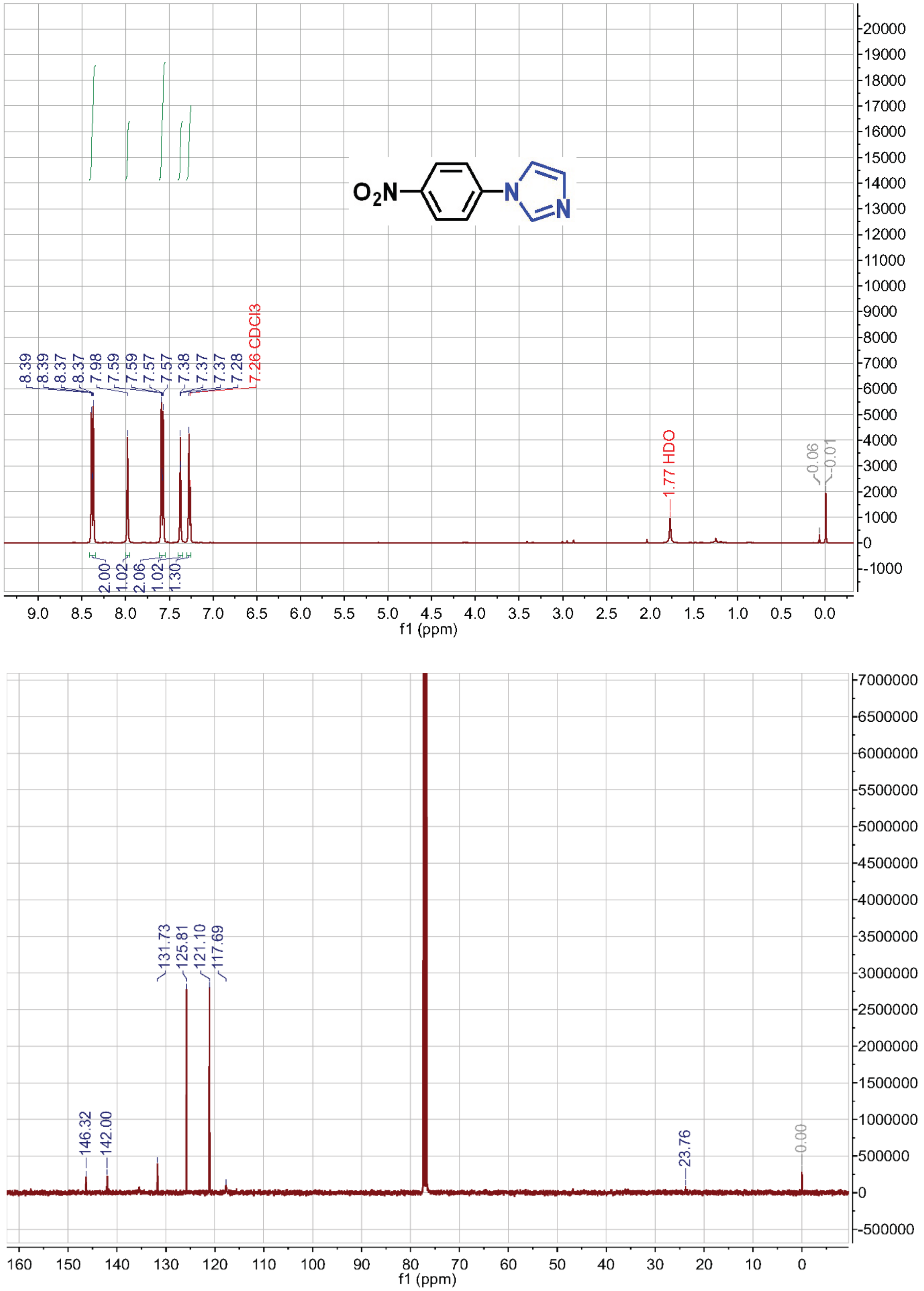

Supplementary Figure 8. ^{1}H MR and ^{13}C NMR spectra of 1-(4-Nitrophenyl)-1H-imidazole, Scheme 1, **5**.

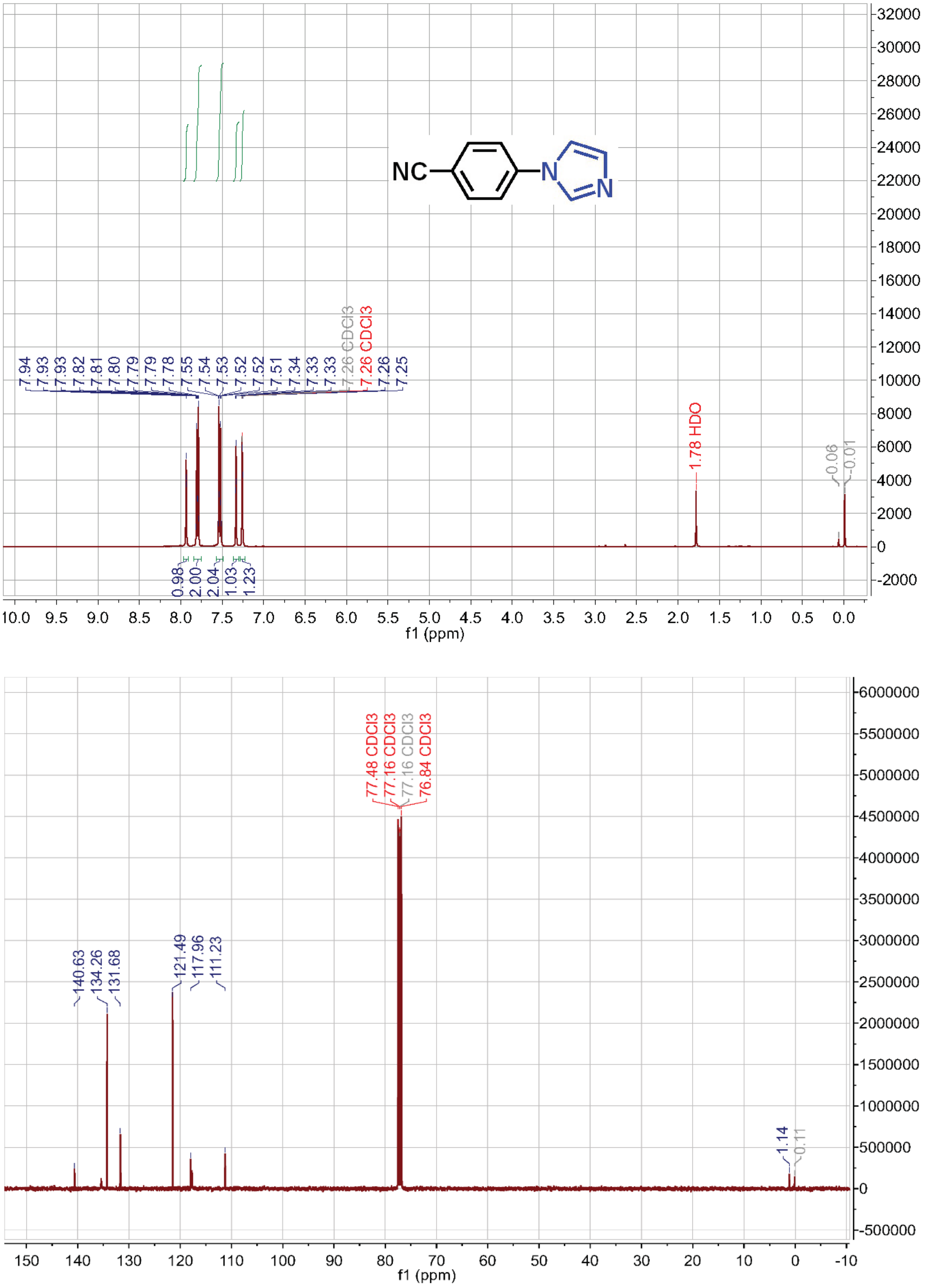

Supplementary Figure 9. ^{1}H MR and ^{13}C NMR spectra of 4-Imidazole-1-yl-benzonitrile, Scheme 1, **6**.

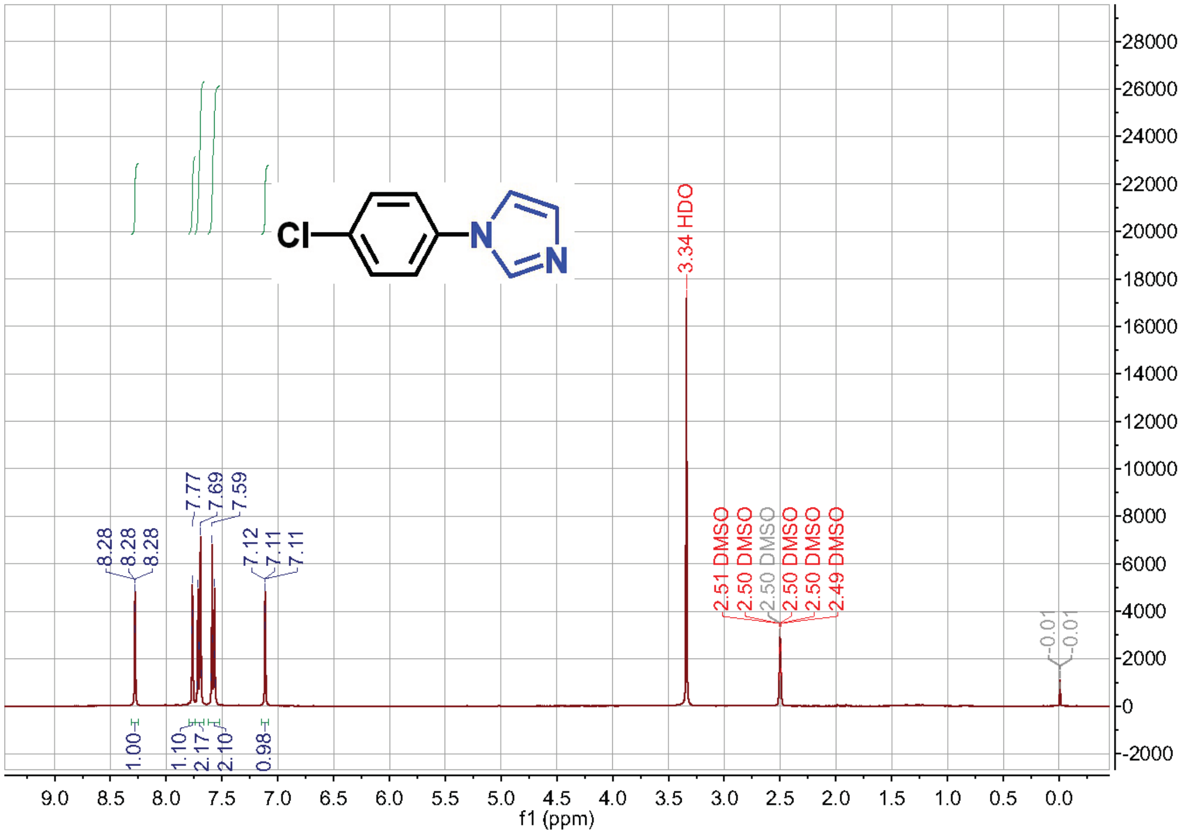

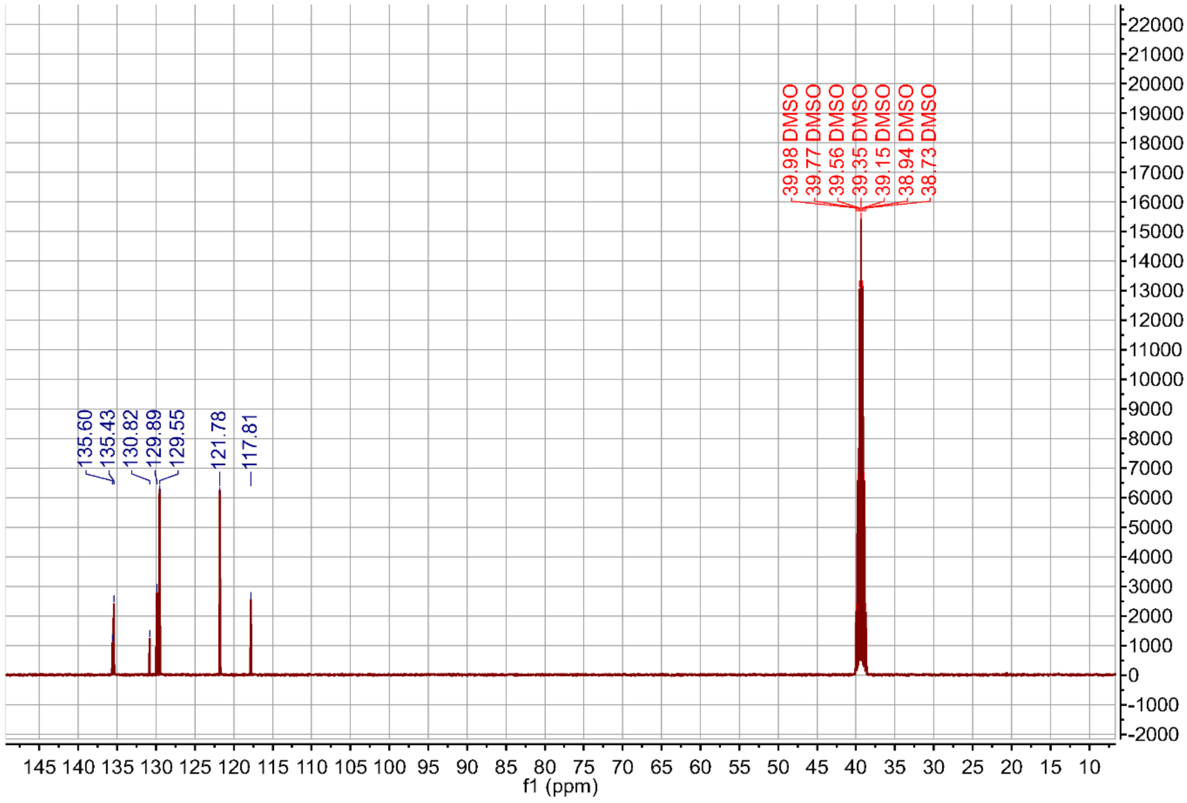

Supplementary Figure 10. ^{1}H MR and ^{13}C NMR spectra of 1-(4-Chloro-phenyl)-1H-imidazole, Scheme 1, **7**.

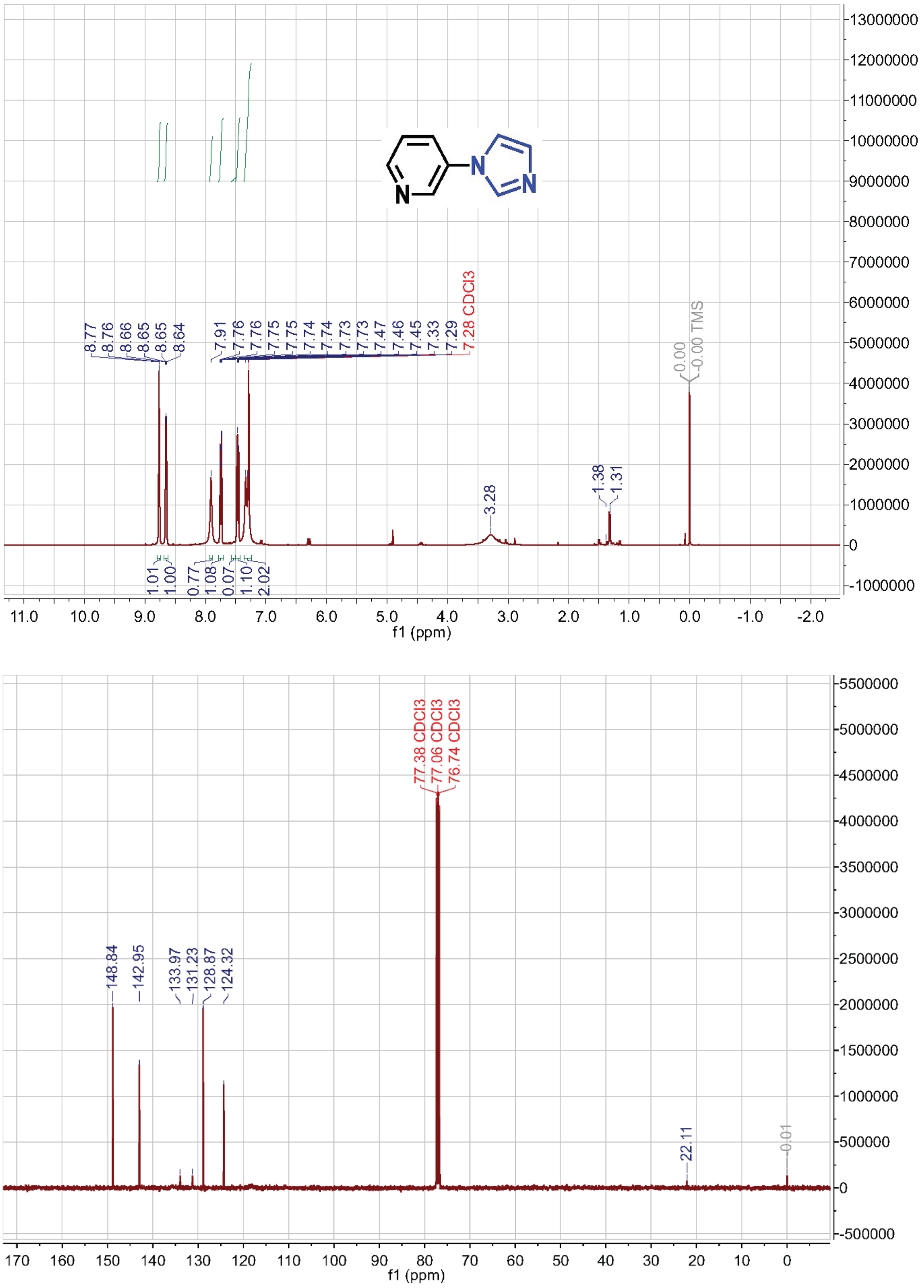

Supplementary Figure 11. ^{1}H MR and ^{13}C NMR spectra of 3-(1H-imidazol-1-yl)pyridine, Scheme 1, **8**.

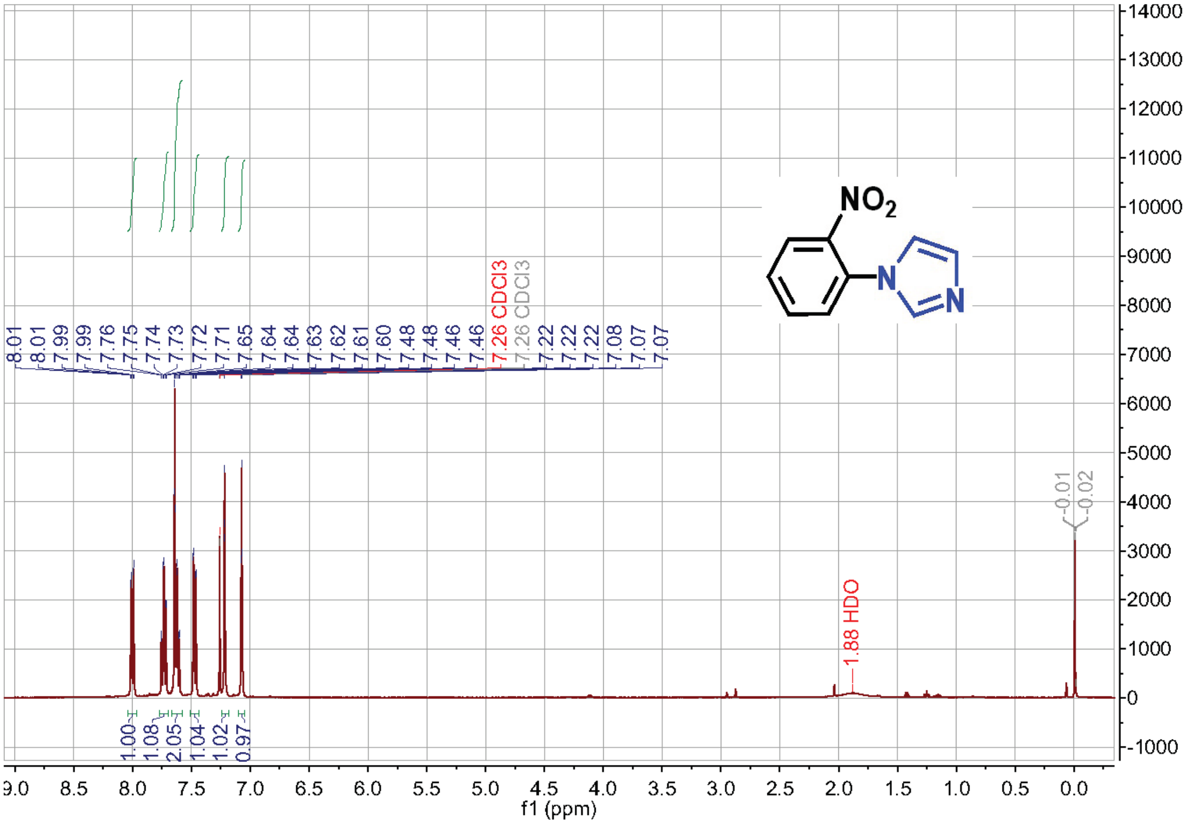

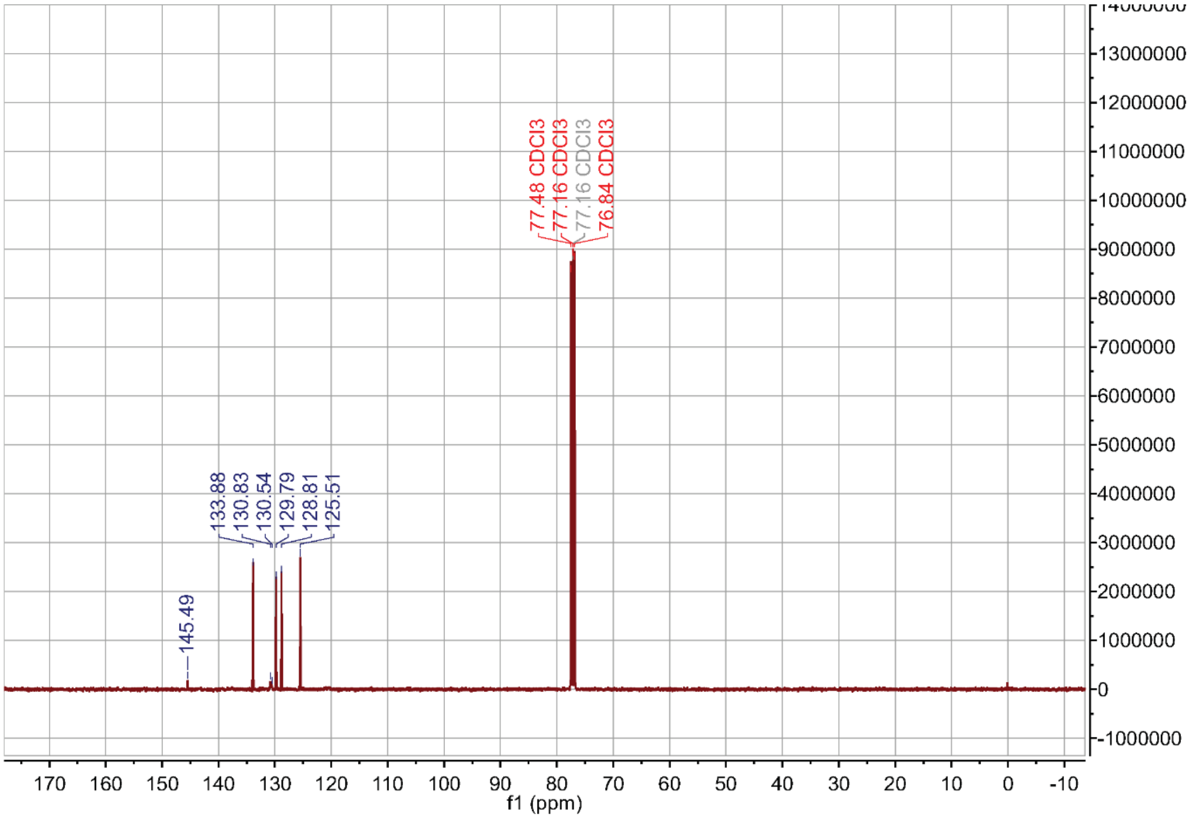

Supplementary Figure 12. ^{1}H MR and ^{13}C NMR spectra of 1-(2-Nitrophenyl)-1H-imidazole, Scheme 1, **9**.

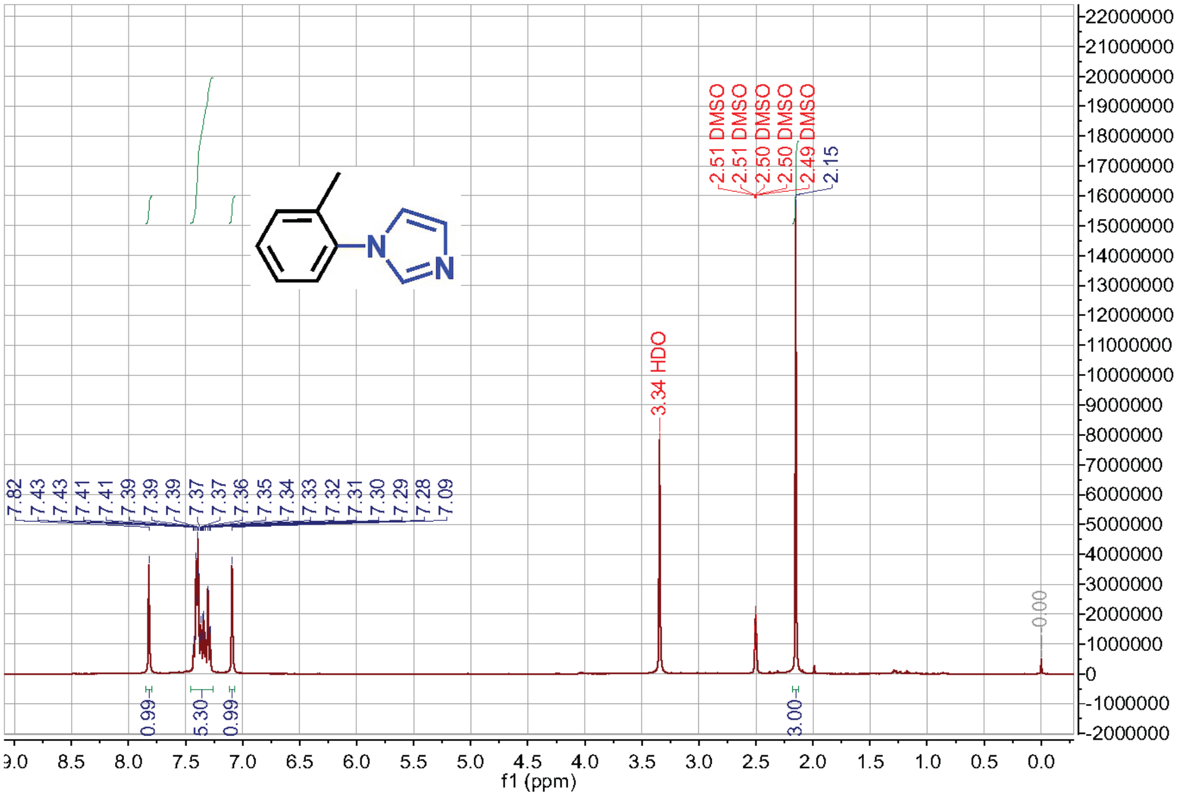

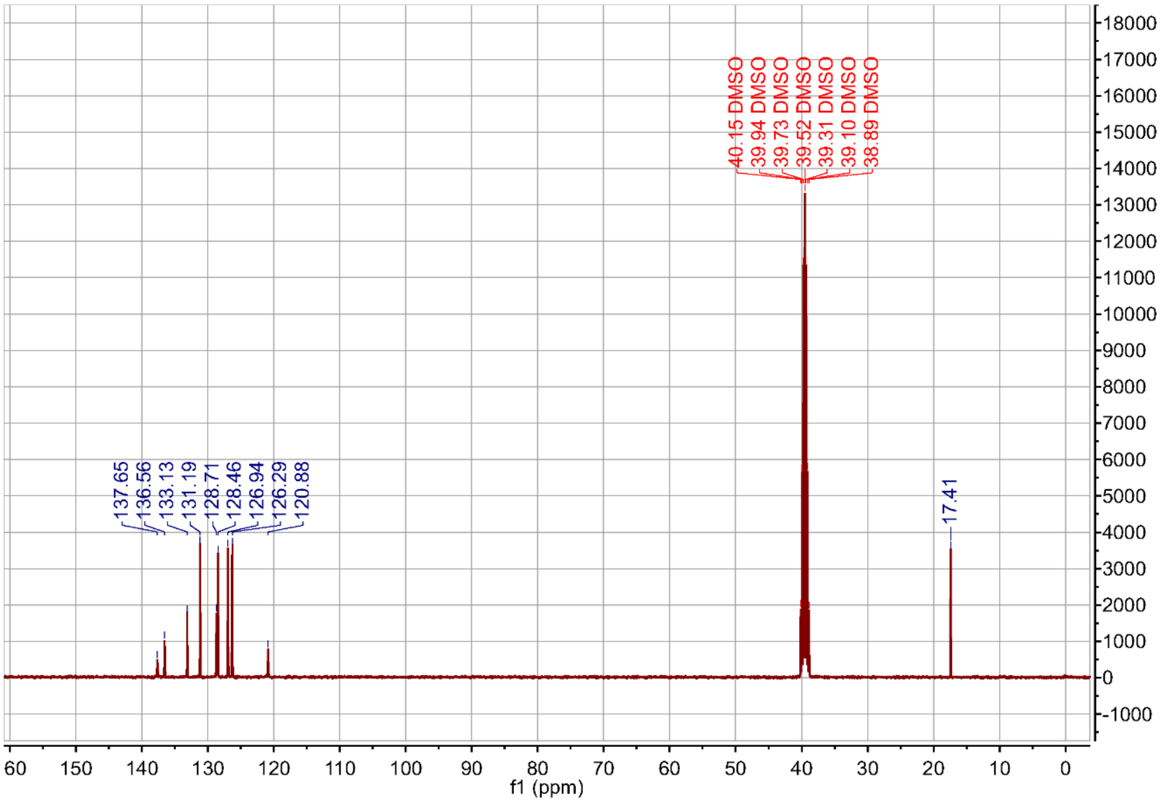

Supplementary Figure 13. ^{1}H MR and ^{13}C NMR spectra of 1-(2-Methylphenyl)-1H-imidazole, Scheme 1, **10**.

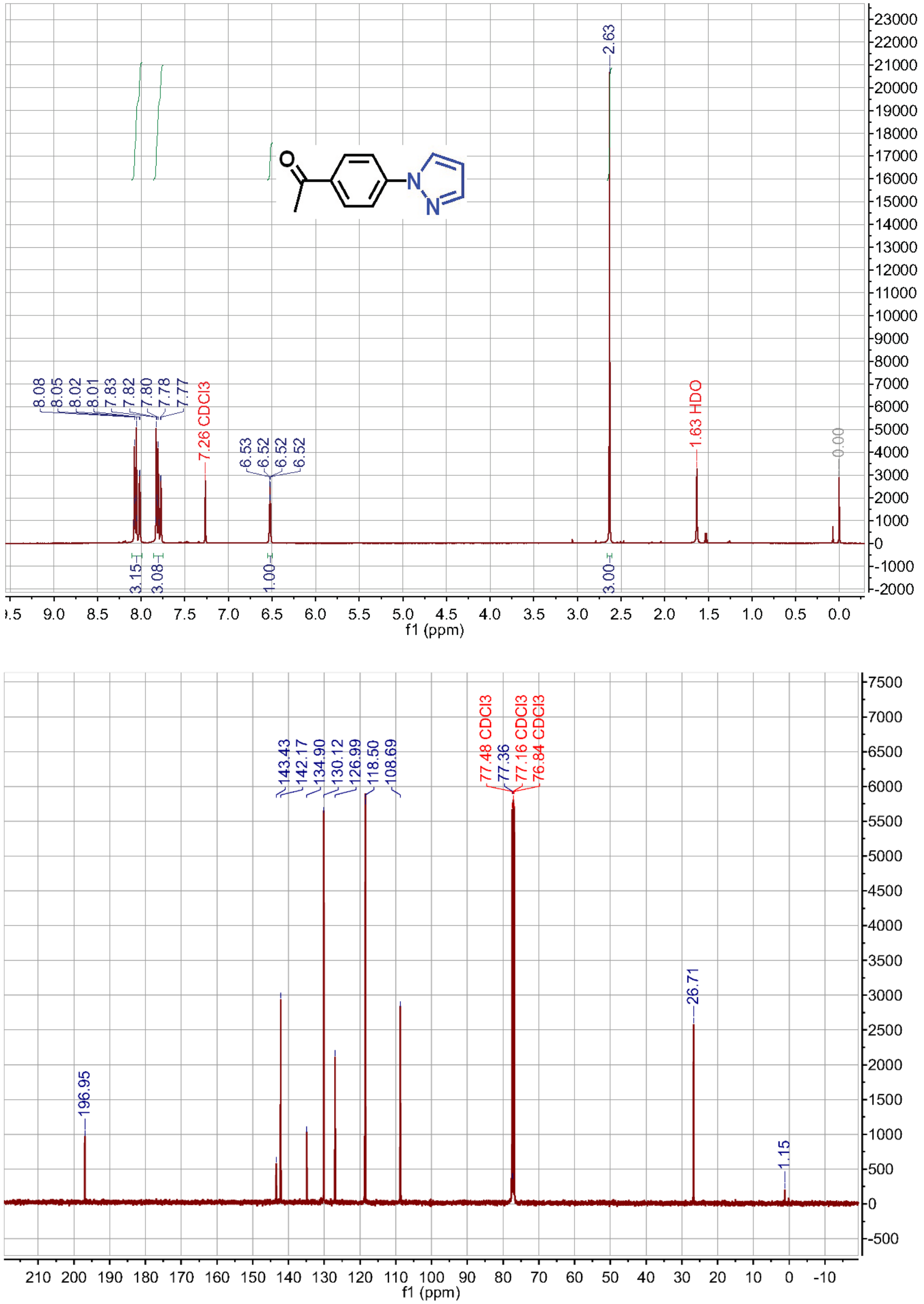

Supplementary Figure 14. ^{1}H MR and ^{13}C NMR spectra of 1-(4-(1H-pyrazol-1-yl)phenyl)ethan-1-one, Scheme 1, **13**.

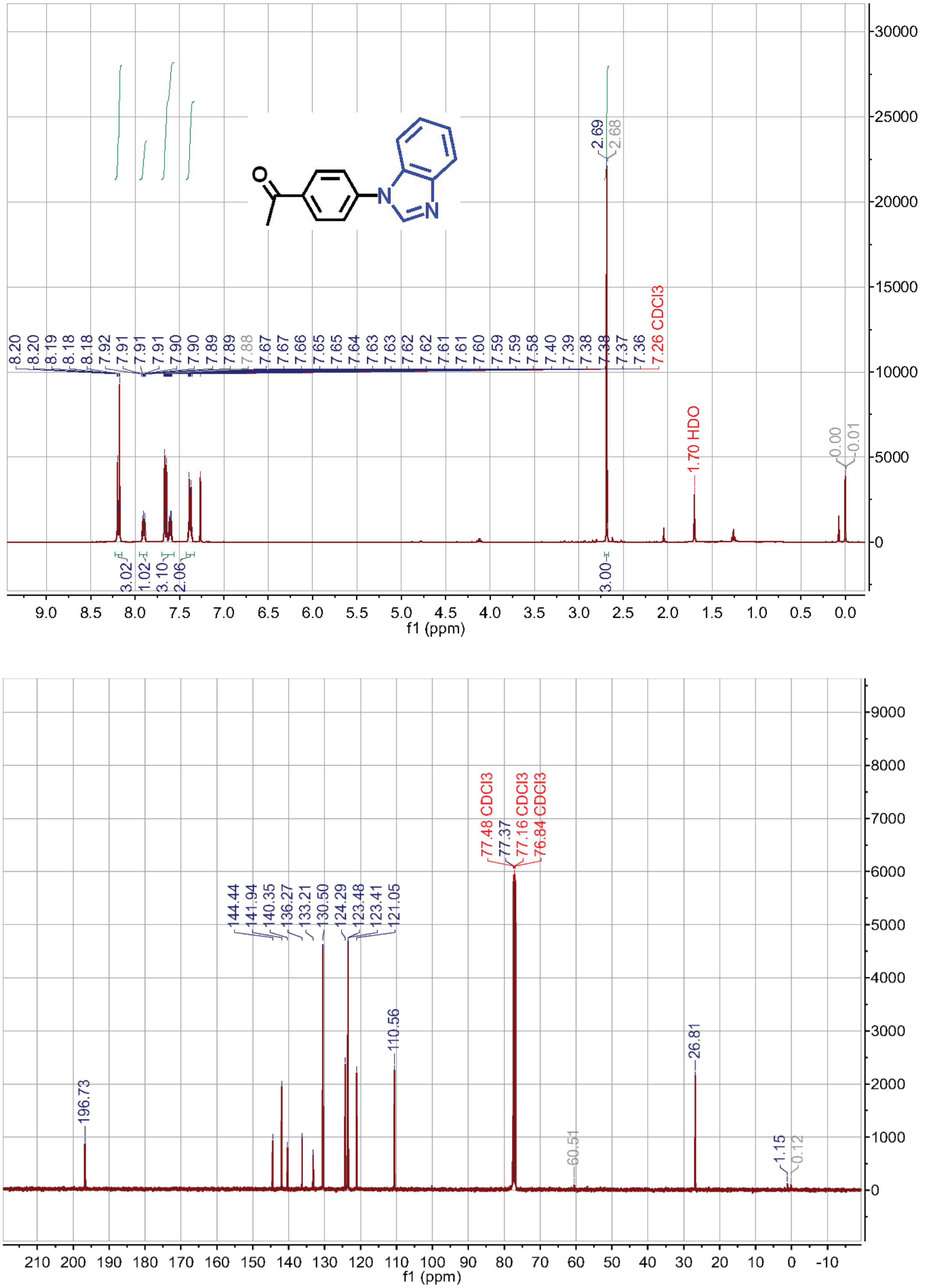

Supplementary Figure 15. ^{1}H MR and ^{13}C NMR spectra of 1-(4-(1H-benzo[d]imidazol-1-yl)phenyl)ethan-1-one, Scheme 1, **14**.

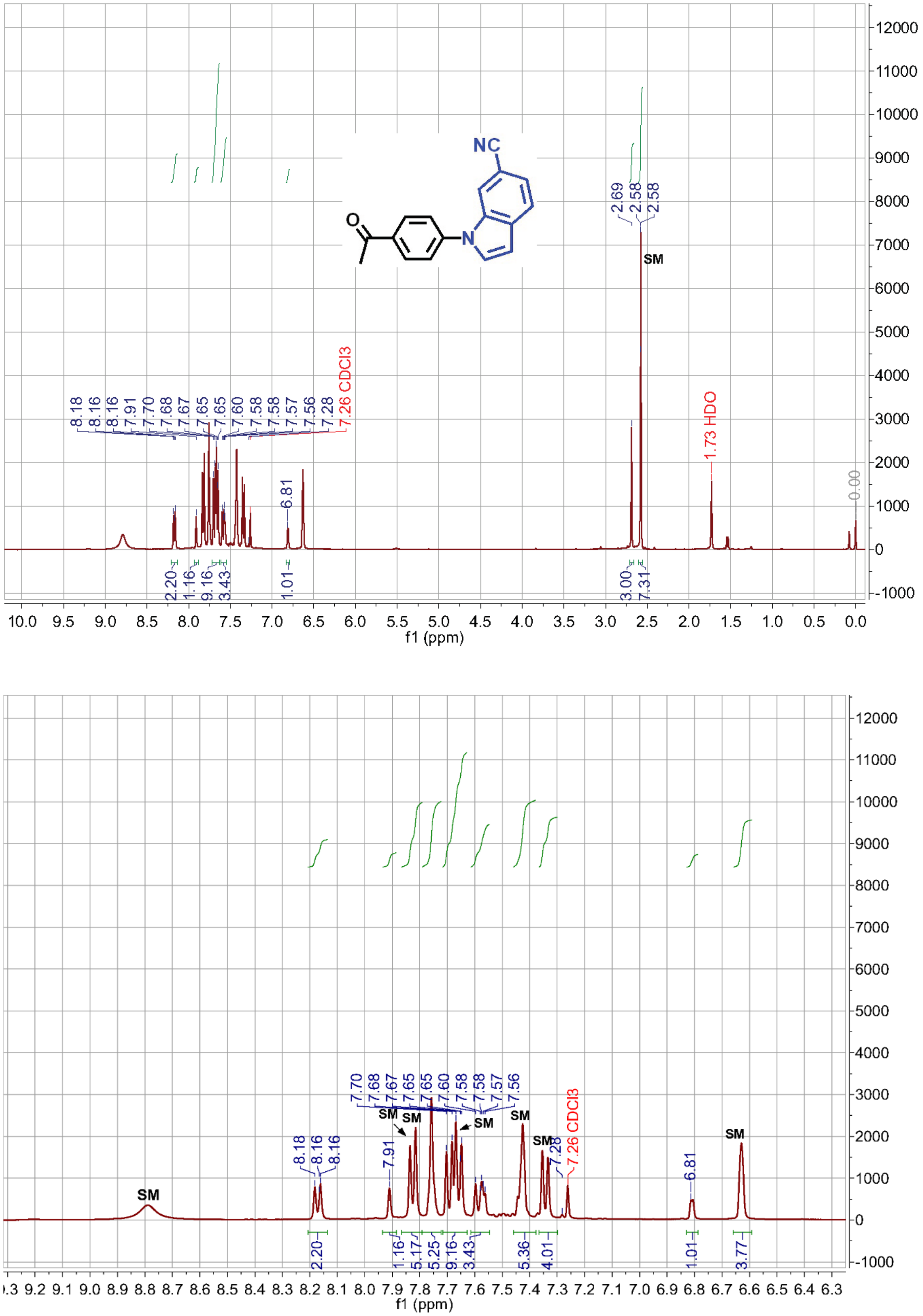

Supplementary Figure 16. ^{1}H MR spectrum of 1-(4-acetylphenyl)-1H-indole-6-carbonitrile, Scheme 1, **15** (top) and a zoomed-in aromatic region of the ^{1}H NMR spectrum (bottom).

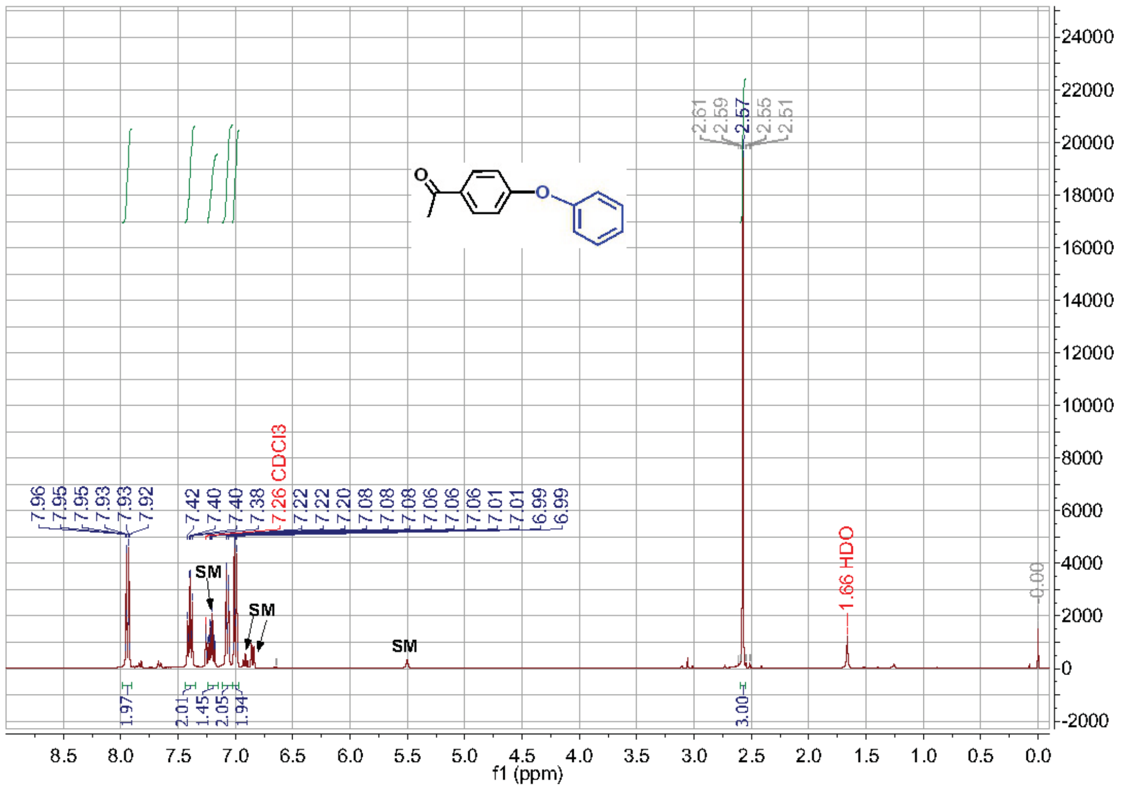

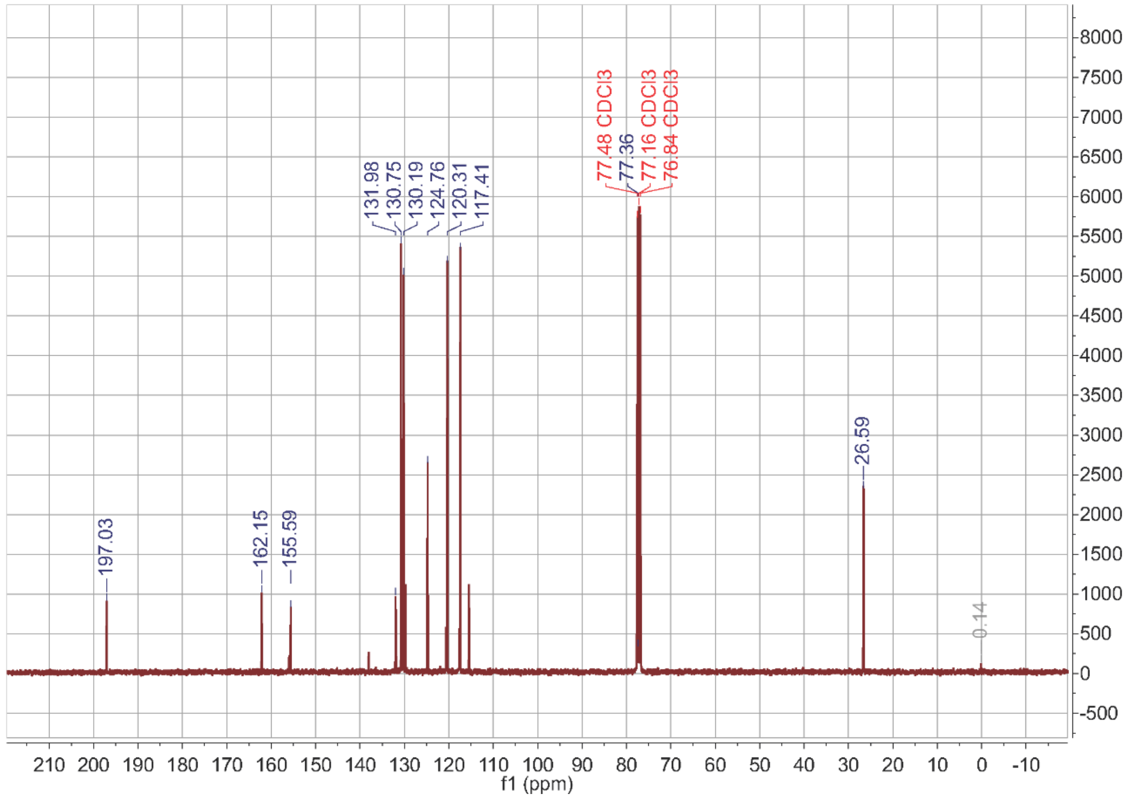

Supplementary Figure 17. ^{1}H MR and ^{13}C NMR spectra of 1-(4-Phenoxyphenyl)ethan-1-one, Scheme 1, **16**.

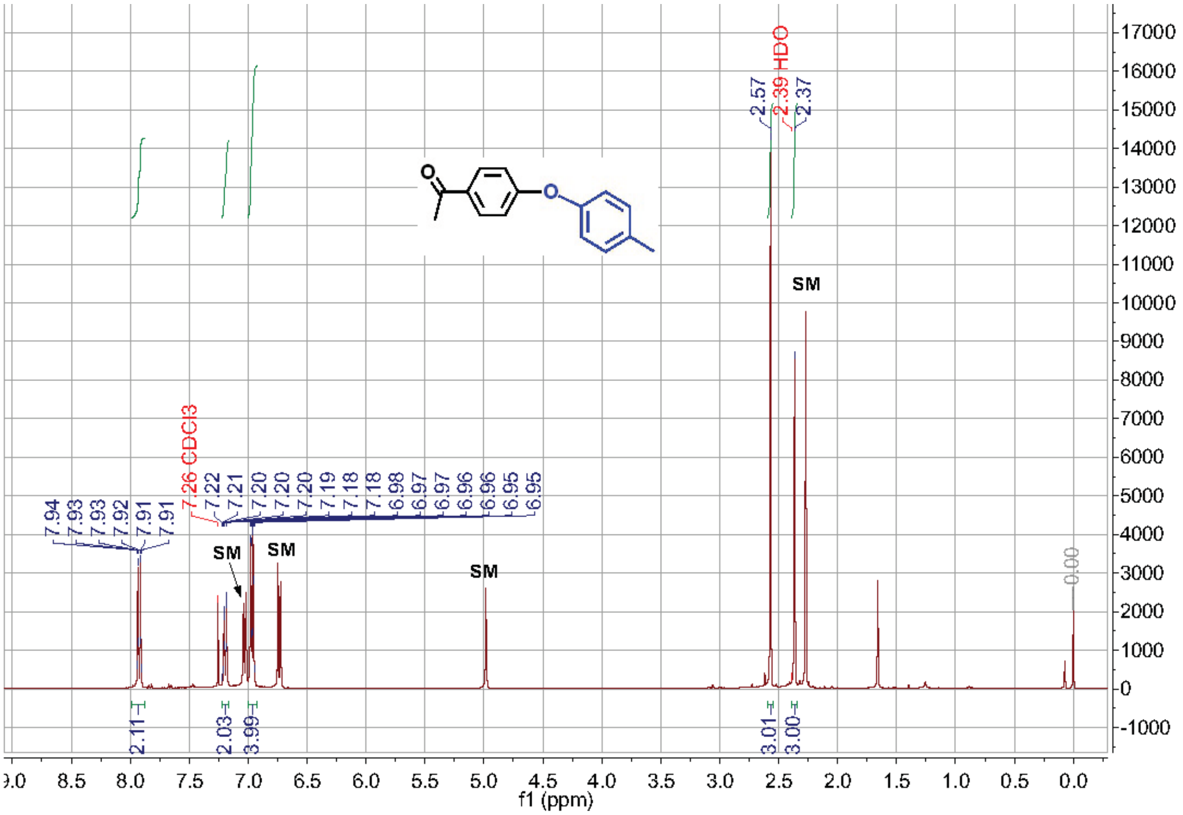

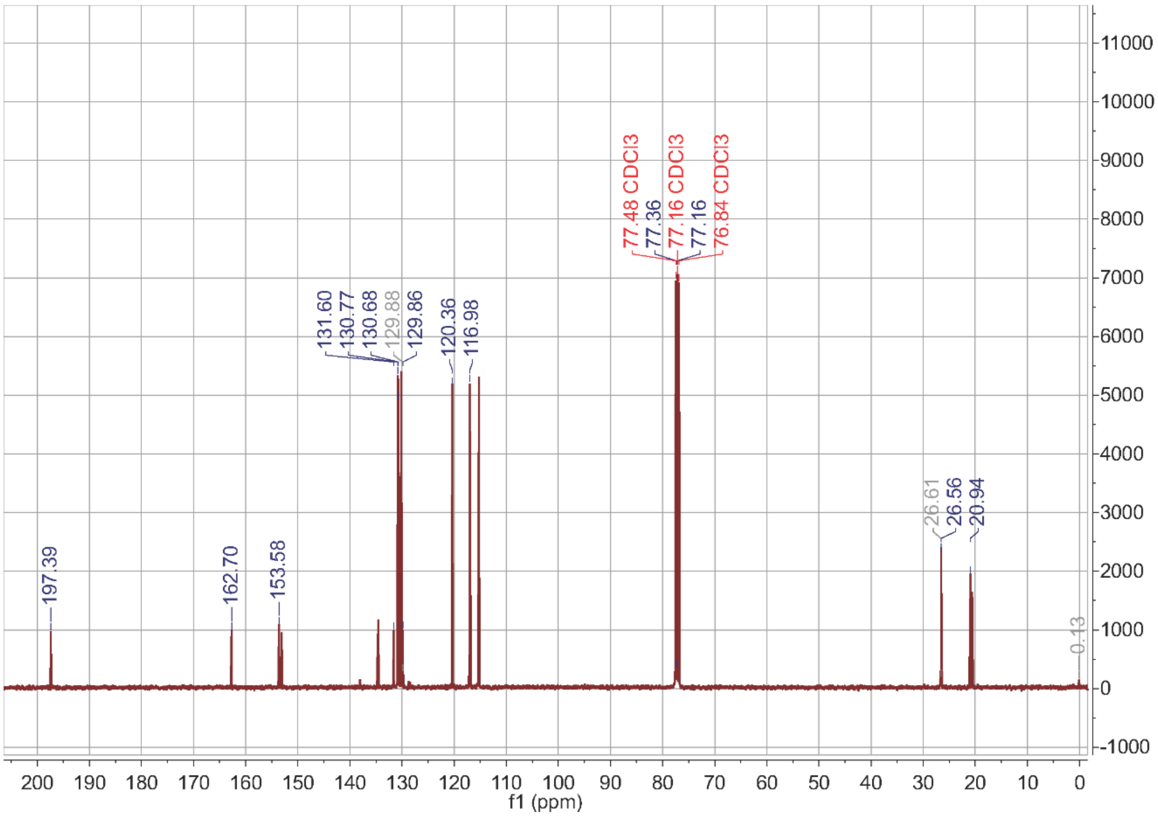

Supplementary Figure 18. ^{1}H MR and ^{13}C NMR spectra of 1-(4-(p-Tolyloxy)phenyl)ethan-1-one, Scheme 1, **17**.

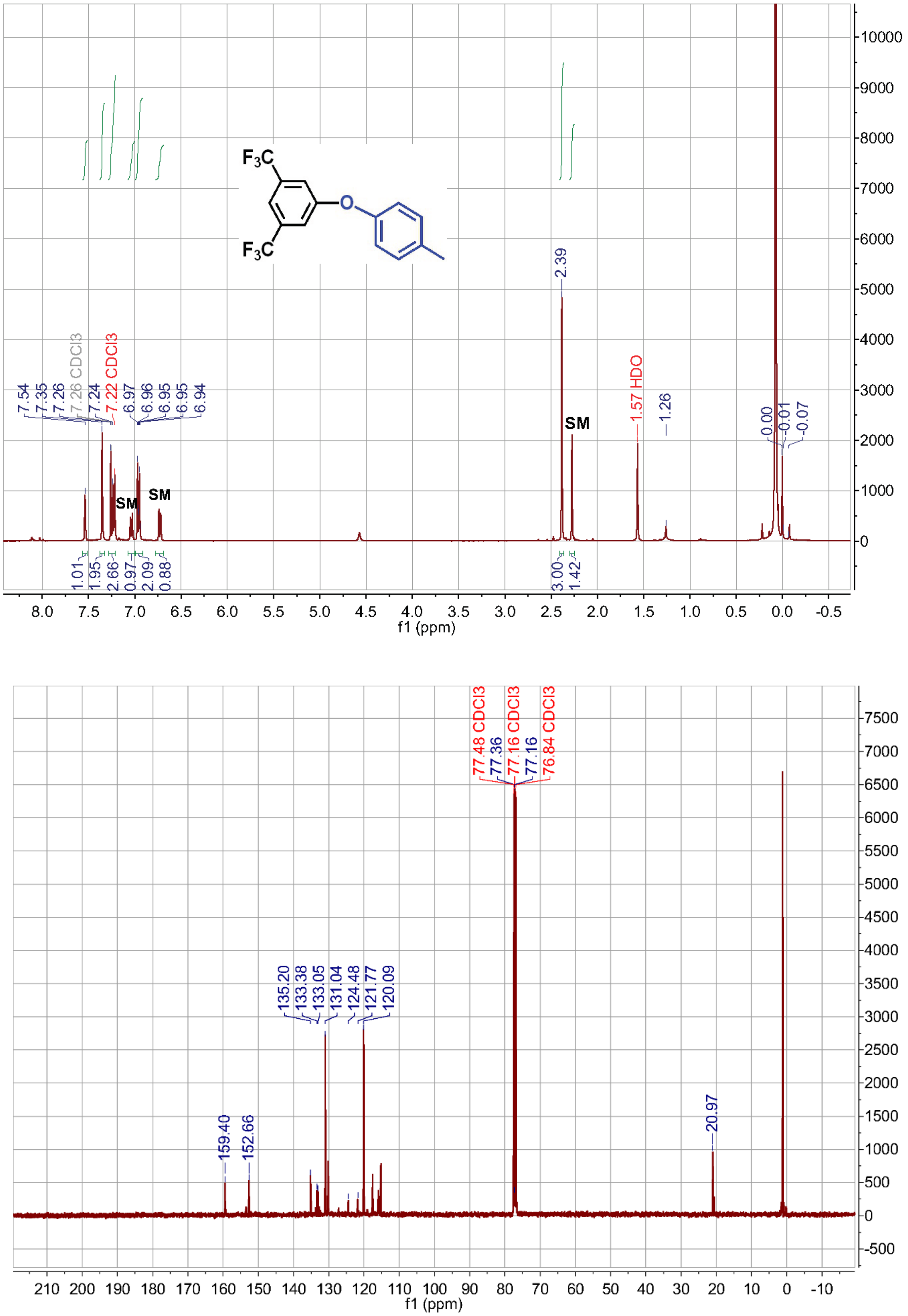

Supplementary Figure 19. ^{1}H MR and ^{13}C NMR spectra of 1-(p-Tolyloxy)-3,5-bis(trifluoromethyl)benzene, Scheme 1, **18**.
